# Screening of ligands for the Ullmann synthesis of electron-rich diaryl ethers

**DOI:** 10.3762/bjoc.8.122

**Published:** 2012-07-17

**Authors:** Nicola Otto, Till Opatz

**Affiliations:** 1 Institute of Organic Chemistry, University of Mainz, Duesbergweg 10–14, 55128 Mainz, Germany

**Keywords:** catalysis, C–O bond formation, diaryl ethers, nucleophilic aromatic substitution, Ullmann-type coupling

## Abstract

In the search for new ligands for the Ullmann diaryl ether synthesis, permitting the coupling of electron-rich aryl bromides at relatively low temperatures, 56 structurally diverse multidentate ligands were screened in a model system that uses copper iodide in acetonitrile with potassium phosphate as the base. The ligands differed largely in their performance, but no privileged structural class could be identified.

## Introduction

**Figure 1:**
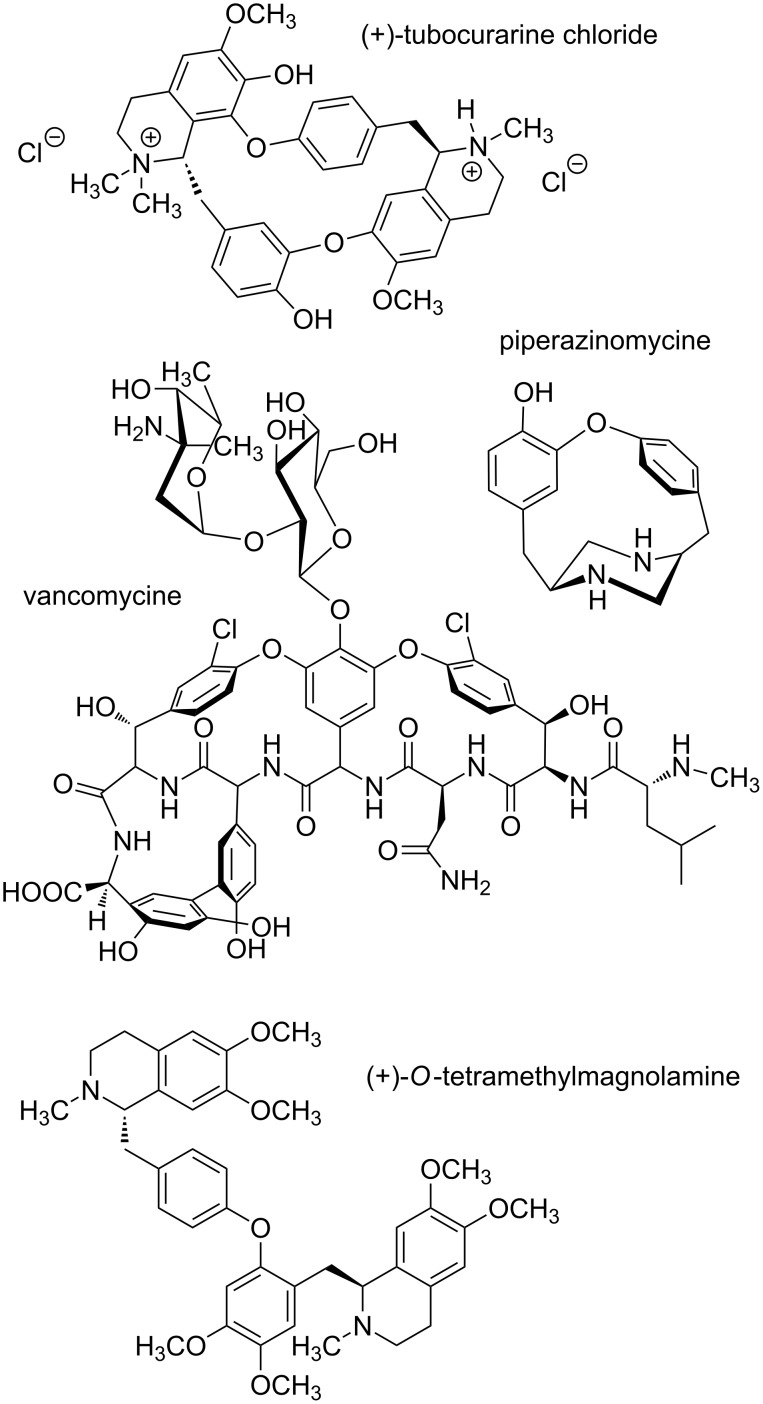
Selected examples of bioactive natural diaryl ethers.

The diaryl ether linkage is a common structural motif encountered in numerous classes of natural products. Moreover, various diaryl ethers have been shown to possess antibacterial, anti-inflammatory, antifungal and herbicidal activity, rendering this compound class attractive for pharmaceutical and agrochemical research [1–2]. Prominent examples of bioactive natural representatives include the glycopeptide antibiotic vancomycin [3–4], the bisbenzylisoquinolines tubocurarine [5] and tetramethylmagnolamine [6], and the antifungal diamine piperazinomycin [7] (Figure 1).

A straightforward method for the formation of diaryl ethers is the Cu-catalyzed coupling of aryl halides with phenols, first reported by Fritz Ullmann in 1903 [8–9]. However, the classical protocol suffers from considerable limitations due to the use of stoichiometric or superstoichiometric amounts of copper powder and typically requires high reaction temperatures (≈200 °C), thus tolerating only a limited number of functional groups. Alternatively, C–O bond formation can be accomplished by efficient Pd-catalyzed arylations of phenols developed by Buchwald [10–11] and Hartwig [12–13] in the 1990s. Nevertheless, the use of this expensive metal in combination with sensitive and costly phosphine ligands limits the use of palladium catalysis for industrial-scale applications and economic aspects have led to a renaissance of the Cu-catalyzed reaction in recent years [14]. It is known that certain additives, such as *N*,*N*- and *N*,*O*-chelating ligands, accelerate the Ullmann diaryl ether synthesis and permit a considerable reduction of the reaction temperature [15–17]. Successful approaches towards a mild coupling were, e.g., developed by Taillefer and Buchwald in 2003 and 2004 who used multidentate ligands and a 5–10 mol % catalyst loading at 90–110 °C [18–20]. However, the majority of ligands reported in the literature to date exhibit a limited substrate scope, and application of the methodology to more complex molecules is still challenging.

For the synthesis of alkaloids containing the diaryl ether linkage, we searched for efficient ligands for the Ullmann coupling of electron-rich aryl bromides as the substrates and performed a ligand screening using the model system 4-bromoanisole/4-methoxyphenol. To the best of our knowledge, this represents the most diverse ligand set investigated for a similar purpose so far [21].

## Results and Discussion

**Figure 2:**
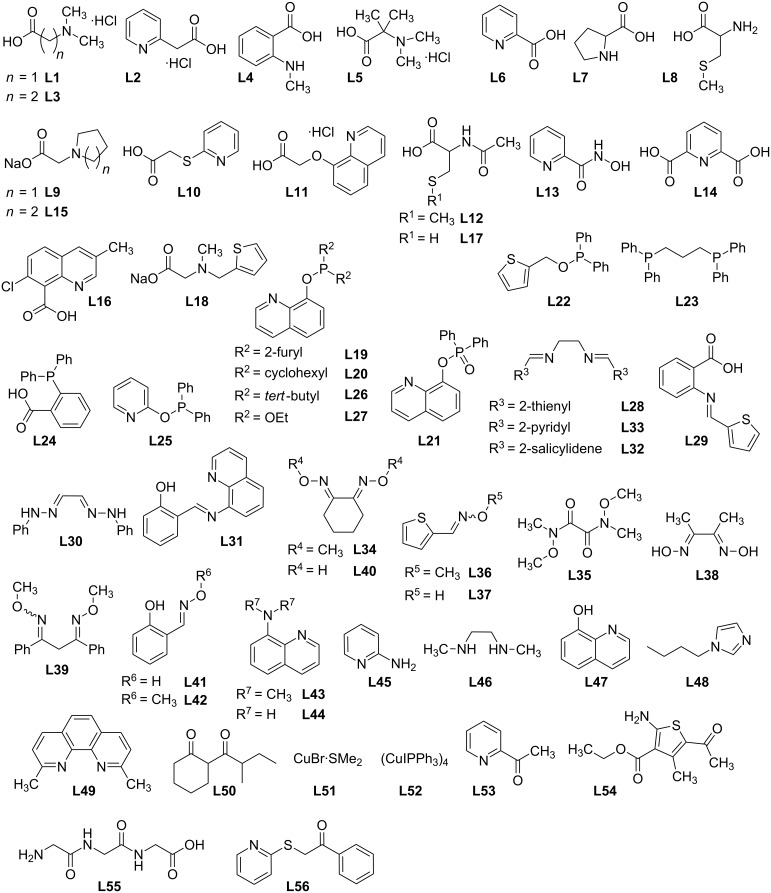
Ligands that were subjected to the ligand screening.

A set of 56 multidentate ligands with different chelating functionalities and bite angles, belonging to the structural classes of amino acids, acetic acid derivatives, phosphinites, phosphonates, imines, diimines, oximes, oxime ethers and diketones were selected for the screening (Figure 2). Several of these compounds have been employed in diaryl ether syntheses before [22–24].

As a starting point, *N*,*N*-dimethylglycine (**L1**) introduced by Ma et al. [22,25] in combination with copper iodide as the Cu-source was chosen, as this ligand has been successfully applied by us in a synthesis of two dimeric benzylisoquinoline alkaloids [26]. This model system was optimized with respect to the influence of different bases, molecular sieves, solvents, and temperatures (Table 1).

**Table 1:** Effect of base, molecular sieves and solvent on the coupling of 4-bromoanisole and 4-methoxyphenol with **L1**.^a^

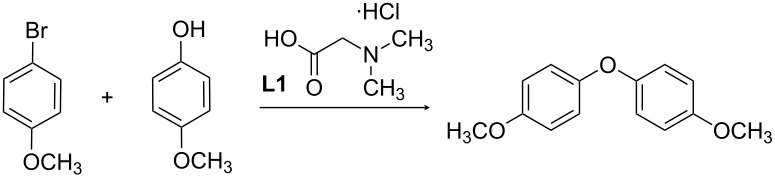					

entry	base	drying agent	CuI (mol %)	solvent	ratio of 4-bromoanisole/product

1	Cs_2_CO_3_	–	10	toluene^b^	no conversion^c^
2	Cs_2_CO_3_	MS 4 Å	5	toluene^b^	conversion^c^
3	Cs_2_CO_3_	MS 4 Å	10	toluene^b^	conversion^c^
4	Cs_2_CO_3_	MgSO_4_	10	toluene^b^	traces^c^
5	K_3_PO_4_	–	10	MeCN^d^	8:1^e^
6	K_3_PO_4_	–	10	1,4-dioxane^f^	9:1^e^
7	K_3_PO_4_	–	10	toluene^b^	2:1^e^

^a^Reaction conditions: base (2.0 equiv), CuI (10 mol %), **L1** (10 mol %), 4-methoxyphenol (1.00 mmol, 1.0 equiv), 4-bromoanisole (1.00 mmol, 1.0 equiv), solvent (0.6 mL), argon atmosphere; ^b^110 °C; ^c^judged by TLC; ^d^80 °C; ^e^determined by ^1^H NMR; ^f^100 °C.

It turned out that caesium carbonate as a base without addition of molecular sieves did not result in any diaryl ether formation, presumably due to the formation of water inactivating the base. With chemical drying agents such as magnesium sulfate, only trace amounts of the product were obtained. In contrast, potassium phosphate proved to be a more suitable base for diaryl ether formation and did not require the addition of molecular sieves. After screening of different solvents, the combination of copper(I) iodide (10 mol %), *N*,*N*-dimethylglycine **L1** (10 mol %) as the ligand, potassium phosphate (2.0 equiv) as the base and acetonitrile as solvent (80 °C) was identified as an efficient system for the coupling of the above-mentioned starting materials (1.0 mmol each in 0.6 mL). Although the substrate conversion in acetonitrile at 80 °C was lower than in toluene at 110 °C (Table 1, entries 5 and 7), acetonitrile was selected as the solvent for the ligand screening in order to be able to compare ligands that give higher conversions than **L1** and to get a better impression of the respective reaction rates. The substrate conversion of the screening reactions was monitored and determined by ^1^H NMR spectroscopy at specific time intervals (2 h 15 min and 24 h).

**Figure 3:**
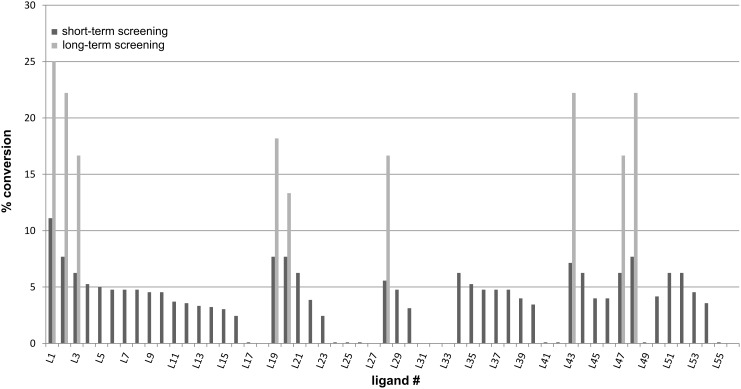
Conversions of the model reaction versus the ligand numbers. For structures of the ligands refer to Figure 2.

In the short-term screening, promising results were obtained with *N*,*P*-chelating phosphinite ligands such as **L19** and **L20**, amino acid-derived *N*,*O*-ligands such as **L2** and **L3** and *N*,*O*- and *N*,*N*-ligands with a rigid backbone such as 8-aminoquinoline (**L44**) and 8-hydroxyquinoline (**L47**) (Table 2 and Figure 3). These ligands have been reported to be effective in the Cu-catalyzed diaryl ether synthesis before [21,27–29].

**Table 2:** Short-term screening.^a^

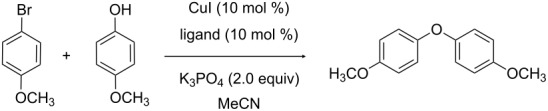					

entry	ligand	ratio 4-bromoanisole/product^b^	entry	ligand	ratio 4-bromoanisole/product^b^

1	**L1**	8:1	29	**L29**	20:1
2	**L2**	12:1	30	**L30**	31:1
3	**L3**	15:1	31	**L31**	no conversion
4	**L4**	18:1	32	**L32**	no conversion
5	**L5**	19:1	33	**L33**	no conversion
6	**L6**	20:1	34	**L34^e^**	15:1
7	**L7**	20:1	35	**L35**	18:1
8	**L8**	20:1	36	**L36**	20:1
9	**L9**	21:1	37	**L37**	20:1
10	**L10**	21:1	38	**L38**	20:1
11	**L11**	26:1	39	**L39**	24:1
12	**L12**	27:1	40	**L40^f^**	28:1
13	**L13**	29:1	41	**L41**	traces
14	**L14**	30:1	42	**L42**	traces
15	**L15**	32:1	43	**L43**	13:1
16	**L16^c^**	40:1	44	**L44**	15:1
17	**L17^d^**	traces	45	**L45**	24:1
18	**L18**	no conversion	46	**L46**	24:1
19	**L19**	12:1	47	**L47**	15:1
20	**L20**	12:1	48	**L48**	12:1
21	**L21**	15:1	49	**L49**	traces
22	**L22**	25:1	50	**L50**	23:1
23	**L23**	40:1	51	**L51^g^**	15:1
24	**L24**	traces	52	**L52^g^**	15:1
25	**L25**	traces	53	**L53**	21:1
26	**L26**	traces	54	**L54**	27:1
27	**L27**	no conversion	55	**L55**	traces
28	**L28**	17:1	56	**L56**	no conversion

^a^reaction conditions: K_3_PO_4_ (2.0 equiv), CuI (10 mol %), ligand (10 mol %), 4-methoxyphenol (1.00 mmol, 1.0 equiv), 4-bromoanisole (1.00 mmol, 1.0 equiv), MeCN (3 mL), 80 °C, argon atmosphere; ^b^determined by ^1^H NMR; ^c^time: 2 h; ^d^time: 2 h 35 min; ^e^time: 3 h; ^f^time: 2 h 40 min; ^g^time: 2 h 25 min.

A disadvantage of ligands with free amino or hydroxy groups, such as **L44**, is that they themselves can act as substrates in the Ullmann-type coupling, which may result in the formation of undesired side products and a loss in catalyst performance. To avoid this complication, the *N*,*N*-dimethylated derivative **L43** was synthesized and turned out to exhibit an improved catalytic activity compared to **L44**. Since **L1** showed high catalytic activity, its 2,2-dimethylated analogue **L5** and the structurally related pyrrolidino- and piperidinoacetic acids **L9** and **L15** were tested, but even these minor modifications of the ligand structure led to a drastically decreased catalytic activity.

Interestingly, not only the *N*,*P*-chelating phosphinite ligands **L19** and **L20** but also the *N*,*O*-chelating phosphonate ligand **L21** showed a significant catalytic activity. From the group of diimine ligands, **L28** [30] gave the best results. It should be noted that also in this class, structurally related ligands showed drastically different catalytic activities in the screening, as can be seen by comparison of **L28**, **L32**, and **L33**. Furthermore, oxime ethers, which, to the best of our knowledge, represent a new class of ligands for Ullmann-type couplings, were also screened in the model reaction. All tested oxime ethers and oximes showed catalytic activity, although the substrate conversion was lower compared to the amino acid-derived ligands. The catalytic activities of the oximes did not, however, differ from those of the oxime ethers. On the other hand, the salicyl aldehyde-derived oxime ether and oxime ligands [31] showed only poor substrate conversions in comparison to other classes of multidentate ligands. **L48** also proved to possess a high catalytic activity, which is consistent with the results reported by Beller et al. [32] who used the ligand for the synthesis of various diaryl ethers. In the short-term screening, none of the tested ligands showed a higher substrate conversion than **L1** did.

The most promising ligands of the short-term screening were subsequently subjected to a long-term screening (24 h) to examine their thermal and chemical stability (Table 3, Figure 3).

**Table 3:** Long-term screening.^a^

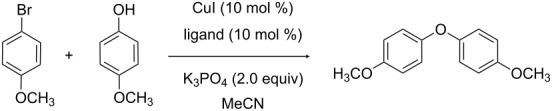							

entry	ligand	time	ratio^b^	entry	ligand	time	ratio^b^

1	**L1**	2 h 15 min 24 h	8:1 3:1	5	**L20**	2 h 15 min 24 h	12:1 6.5:1
2	**L2**	2 h 15 min 24 h	12:1 3.5:1	6	**L28**	2 h 15 min 3 h 15 min 24 h	17:1 9:1 5:1
3	**L3**	2 h 15 min 24 h	15:1 5:1	7	**L43**	2 h 15 min 24 h	13:1 3.5:1
4	**L19**	2 h 15 min 24 h	12:1 4.5:1	8	**L47**	2 h 15 min 24 h	15:1 5:1
				9	**L48**	2 h 15 min 24 h	12:1 3.5:1

^a^Reaction conditions: K_3_PO_4_ (2.0 equiv), CuI (10 mol %), ligand (10 mol %), 4-methoxyphenol (1.00 mmol, 1.0 equiv), 4-bromoanisole (1.00 mmol, 1.0 equiv), MeCN (3 mL), 80 °C; ^b^ratio of 4-bromoanisole to product, determined by ^1^H NMR.

In the long-term screening the ligands **L2**, **L43** and **L48** showed promising results since their substrate conversions were only slightly lower than that of **L1**, which again was unsurpassed. The phosphinite ligands **L19** and **L20**, which showed comparable catalytic activities to **L2**, **L43** and **L48** in the short-term screening, in comparison gave lower substrate conversions in the long-term screening. This decrease of catalytic activity may be due to oxidation and subsequent hydrolysis of the air- and water-sensitive phosphinites to their corresponding phosphinic acids and 8-hydroxyquinoline (**L47**), which showed a comparable catalytic activity. It was found that the conversion within a specific time interval decreased over time stronger than anticipated, and finally the reaction ceased. The stagnation may be due to the formation of traces of water in the reaction mixture or formation of a catalytically less active copper(I)–bromide complex from liberated bromide ions. Another alternative may be the decomposition of the ligands.

## Conclusion

A collection of 56 multidentate ligands were screened in a model system for the Ullmann diaryl ether synthesis of electron-rich phenols and aryl bromides. Structurally diverse ligands showed a catalytic activity in the coupling, but none of the ligands showed a higher catalytic activity than *N,N*-dimethylglycine (**L1**). In the long-term screening, the ligands **L2**, **L43** and **L48** were found to exhibit only slightly lower catalytic activity than **L1** in the model reaction. In conclusion, *N*-methylated amino acid-derived *N,O*-ligands, *N,N*-ligands with small bite angles, and *N*-butylimidazole proved to be the most efficient ligands for Ullmann diaryl ether formation under the tested conditions. The synthesized phosphinite-type *P,N*-ligands derived from 8-hydroxyquinoline performed well in the short-term screening but lost catalytic activity over time. While active ligands were found in various structural classes, even relatively small variations of the efficient ligands can lead to a dramatic loss of catalytic activity, which complicates their rational optimization. In general, strongly chelating ligands with four coordinating atoms should be avoided. Coordination of the copper atom by one N- and one N- or O-atom may be a desirable structural motif for the design of new ligands. Remarkably, published ligands used for other C-heteroatom bond-formation reactions showed unexpected low activity in our system.

## Experimental

### General procedure for model reactions of the ligand screening

A dried reaction tube (diameter 1.5 cm) equipped with a stirring bar (length 0.6 cm) is charged with anhydrous K_3_PO_4_ (430 mg, 2.00 mmol, 2.0 equiv) under an argon atmosphere in counterflow. CuI (14 mg, 0.10 mmol, 10 mol %), ligand (0.10 mmol, 10 mol %), 4-methoxyphenol (124 mg, 1.00 mmol, 1.0 equiv), 4-bromoanisole (130 μL, 187 mg, 1.00 mmol, 1.0 equiv) and 1 mL of anhydrous acetonitrile is subsequently added, and the tube is sealed with a septum and equipped with an argon balloon. The reaction mixture is stirred for 30 min at room temperature and is then placed in a heating bath at 80 °C. Samples of 500 μL of the reaction mixture are taken after 2 h 15 min and 24 h. The samples are filtered over Celite and washed with dichloromethane, and the filtrate is evaporated in vacuo. The conversion of starting material is determined by ^1^H NMR spectroscopy.

## Supporting Information

File 1 Experimental procedures and characterization data of ligands and starting materials.

## References

[1] Ley S V, Thomas A W (2003). Angew Chem, Int Ed.

[2] Frlan R, Kikelj D (2006). Synthesis.

[3] Sheldrick G M, Jones P G, Kennard O, Williams D H, Smith G A (1978). Nature.

[4] Nicolaou K C, Mitchell H J, Jain N F, Winssinger N, Hughes R, Bando T (1999). Angew Chem, Int Ed.

[5] Teuscher E, Melzig M F, Lindequist U (2004). Biogene Arzneimittel.

[6] Manske R H F, Holmes H L (1954). The Alkaloids–Chemistry & Physiology.

[7] Tamai S, Kaneda M, Nakamura S (1982). J Antibiot.

[8] Ullmann F (1904). Ber Dtsch Chem Ges.

[9] Ullmann F, Sponagel P (1905). Ber Dtsch Chem Ges.

[10] Aranyos A, Old D W, Kiyomori A, Wolfe J P, Sadighi J P, Buchwald S L (1999). J Am Chem Soc.

[11] Kuwabe S, Torraca K E, Buchwald S L (2001). J Am Chem Soc.

[12] Hartwig J F (1998). Angew Chem, Int Ed.

[13] Mann G, Incarvito C, Rheingold A L, Hartwig J F (1999). J Am Chem Soc.

[14] Kunz K, Scholz U, Ganzer D (2003). Synlett.

[15] Weingarten H (1964). J Org Chem.

[16] Paine A J (1987). J Am Chem Soc.

[17] Capdevielle P, Maumy M (1993). Tetrahedron Lett.

[18] Taillefer M, Cristau H J, Cellier P P, Spindler J F, Ouali A (2003). Relatively low-temperature catalytic procedure for arylation or vinylation of nitrogen-containing nucleophilic compounds. WO Patent.

[19] Taillefer M, Cristau H J, Cellier P P, Spindler J F (2003). Process for arylation, vinylation or alkynylation of nucleophilic compounds, in particular nitrogen-containing nucleophiles. WO Patent.

[20] Buchwald S L, Klapars A, Antilla J C, Job G E, Wolter M, Kwong F Y, Nordmann G, Hennessy E J (2004). Copper-Catalyzed Formation of Carbon-Heteroatom and Carbon-Carbon Bonds. U.S. Patent.

[21] Fagan P J, Hauptman E, Shapiro R, Casalnuovo A (2000). J Am Chem Soc.

[22] Zhang H, Cai Q, Ma D (2005). J Org Chem.

[23] Monnier F, Taillefer M (2009). Angew Chem.

[24] Buck E, Song Z J, Tschaen D, Dormer P G, Volante R P, Reider P J (2002). Org Lett.

[25] Cai Q, Zhu W, Zhang H, Zhang Y, Ma D (2005). Synthesis.

[26] Blank N, Opatz T (2011). J Org Chem.

[27] Liu Y-H, Li G, Yang L-M (2009). Tetrahedron Lett.

[28] Maiti D, Buchwald S L (2010). J Org Chem.

[29] Yamakawa K (1991). Process for Producing Alkoxybenzene Compound and Aryloxybenzene Compound. U.S. Patent.

[30] Hosseinzadeh R, Golchoubian H, Masoudi M (2008). J Chin Chem Soc.

[31] Cristau H-J, Cellier P P, Hamada S, Spindler J-F, Taillefer M (2004). Org Lett.

[32] Schareina T, Zapf A, Cotté A, Müller N, Beller M (2008). Tetrahedron Lett.

