# Supplementary material for: Screening of ligands for the Ullmann synthesis of electron-rich diaryl ethers
Source: Beilstein J Org Chem. 2012 Jul 17;8:1105–11. doi: 10.3762/bjoc.8.122 (PMC3458727; doi:10.3762/bjoc.8.122)
Supplement: File 1 — Experimental procedures and characterization data of ligands and starting materials. [file Beilstein_J_Org_Chem-08-1105-s001.pdf]

Supporting Information

for

Screening of ligands for the Ullmann synthesis of electron-rich diaryl ethers

Nicola Otto and Till Opatz*

Address: Institute of Organic Chemistry, University of Mainz, Duesbergweg 10–14, 55128 Mainz, Germany

Email: Till Opatz* - opatz@uni-mainz.de

* Corresponding author

Synthesis and characterization data of ligands and starting materials

General methods

Solvents were purified according to known literature procedures prior to use. Unless otherwise stated, all starting materials and reagents were purchased from commercial sources and used without further purification. All reactions in anhydrous solvents were performed under argon atmosphere and in oven-dried glassware. For flash column chromatography, Acros Organics silica gel with a particle size of 35–70 µm was used. NMR spectra were recorded on a Bruker AC 300 (300 MHz ^{1}H NMR und 75.5 MHz ^{13}C NMR) and on a Bruker Avance-II 400 (400 MHz ^{1}H NMR and 100.6 MHz ^{13}C NMR, including 2-D spectra). Chemical shifts (δ) are expressed in parts per million (ppm). Coupling constants (*J*) are expressed in hertz (Hz). Chemical shifts are referenced to residual solvents ($CDCl_3$ (^{1}H: δ = 7.26; ^{13}C: δ = 77.16); DMSO-d_6 (^{1}H: δ = 2.50; ^{13}C: δ = 39.52), MeCN-d_3(^{1}H: δ = 1.94; ^{13}C: δ = 1.32, 118.3), D_2O (^{1}H: δ = 4.79), MeOH-d_4 (^{1}H: δ = 3.31; ^{13}C: δ = 49.0), acetone-d_6 (^{1}H: δ = 2.50; ^{13}C: δ = 29.8, 206.3)) [1]. For HPLC-MS and ESI-MS analyses an Agilent Technologies 1200 series with a binary pump system and a diode array detector, which is coupled with a Bruker LCD/MSD trap mass spectrometer, was used. For ionization, an electron spray ion source was employed. Solvents: A (water + 0.1% formic acid), B (acetonitrile + 0.1% formic acid), C (ammonia acetate, adjusted to pH 3 with formic acid). Method A: column: Ascentis Express C_{18}, 2.7 µm particle size (core–shell), length 3 cm, diameter 2.1 mm; temp. column oven: 40 °C; flow: 0.5 mL/min; solvent A and B, gradient: 10% B to 100% B in 3 min. Method B: column: Ascentis Express C_8, particle size 2.7 µm (core–shell), length 3 cm, diameter 2.1 mm; temp. column oven: 40 °C; flow: 1.0 mL/min; solvent B and C, gradient: 20% B to 90% B in 2 min. High-resolution ESI-mass spectra (ESI-HRMS) were analyzed on a Waters QToF-Ultima 3 instrument with a LockSpray interface. Melting points were measured on an A. Krüss Optronic melting-point meter and are uncorrected. IR spectra were recorded on a Jasco 4100 series FT-IR spectrometer with diamond ATR unit. Reaction yields

of the ligand syntheses are not optimized. The spectroscopic data of the known compounds are according to the literature.

4,4'-Dimethoxydiphenyl ether (1)

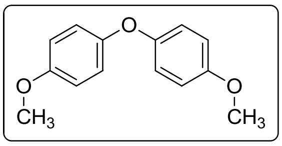

M [$C_{14}H_{14}O_3$]: 230.26 g/mol.

According to a modified procedure of Christau [2] and Ouali [3] et al., in a heat-dried reaction vial with magnetic stirring bar is placed copper iodide (19 mg, 0.1 mmol, 10 mol %), *N*,*N*-dimethylglycine hydrochloride (28 mg, 0.2 mmol, 20 mol %), caesium carbonate (1.30 g, 4.0 mmol, 2.0 equiv), 4 Å molecular sieve (600 mg), 4-methoxyphenol (240 mg, 2.0 mmol, 1.0 equiv), 4-bromoanisole (0.38 mL, 560 mg, 3.0 mmol, 1.5 equiv) and anhydrous toluene (1.2 mL). The reaction vial is capped with a septum and with an argon balloon and is stirred for 6 h at 110 °C. Dichloromethane is added, and the reaction mixture is filtered through Celite. The solvent is evaporated and the residue is purified by flash chromatography on silica gel (cyclohexane/EtOAc = 15:1). The product (0.31 g, 1.35 mmol, 66%) is obtained as colorless crystals.

$\boldsymbol{R_f}$: 0.60 (cyclohexane/EtOAc = 4:1).

mp: 102.5–103.0 °C (cyclohexane/EtOAc) (lit.: 101–103 °C) [4].

^{1}H NMR (300 MHz, $CDCl_3$): δ [ppm] = 6.98–6.90 (AA‘-part of an AA‘BB‘-system, 4H, H2, H6, H2‘, H6‘), 6.88–6.79 (BB‘-part of an AA‘BB‘-system, 4H, H3, H5, H3‘, H5‘), 3.79 (s, 6H, OCH_3).

EA: Anal. calc.: 73.03% C; 6.13% H; found: 73.02% C; 6.31% H.

General procedure A for synthesis of oxime ethers:

According to a modified procedure of MacNevin [5] et al., methoxyamine hydrochloride (1.1 equiv) is suspended in anhydrous methanol. Anhydrous pyridine is added (pyridine/methanol = 1:1) and the aldehyde (1.0 equiv) is added dropwise. The reaction mixture is stirred for 13 h at room temperature. After complete conversion the reaction mixture is taken up in dichloromethane and is washed with 5% citric acid aqueous solution (3 × 50 mL). The combined organic phases are dried over $MgSO_4$ and filtered, and the solvent is evaporated in vacuo. The liquid residue is purified by flash column chromatography on silica gel.

(*Z*)-2- Thiophenecarbaldehyde-*O*-methyloxime (L36)

(*E*)-2-Thiophenecarbaldehyde-*O*-methyloxime (L36a)

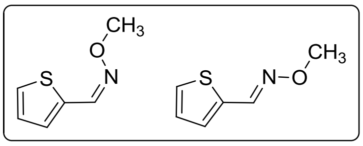

M [C_6H_7NOS]: 141.19 g/mol.

Methoxyamine hydrochloride (410 mg, 4.90 mmol, 1.1 equiv) is suspended in anhydrous methanol (3 mL). Anhydrous pyridine (3 mL) is added and 2-thiophene carbaldehyde (0.42 mL, 500 mg, 4.45 mmol, 1.0 equiv) is added dropwise. The reaction mixture is stirred for 13 h at room temperature. After complete conversion of starting material the reaction mixture is taken up in dichloromethane and is washed with 5% citric acid aqueous solution (3 × 50 mL). The combined organic phases are dried over $MgSO_4$ and filtered, and the solvent is gently evaporated in vacuo (up to 150 mbar, product is volatile). The liquid residue is purified by flash column chromatography on silica gel (cyclohexane/EtOAc = 15:1). The (*E*)-

isomer (197 mg, 31%) and the (*Z*)-isomer are obtained (94 mg, 15%) as a rose oil. In addition, a mixture of both isomers (169 mg, 27%) was obtained.

Total yield: 459 mg (3.25 mmol, 73%).

$\boldsymbol{R}_\mathbf{f}$ ((*E*)-isomer): 0.69 (cyclohexane/EtOAc = 15:1).

$\boldsymbol{R}_\mathbf{f}$ ((*Z*)-isomer): 0.50 (cyclohexane/EtOAc = 15:1).

Isomer 1 (*E*): [6]

^{1}H NMR (300 MHz, $CDCl_3$): δ [ppm] = 8.21 (s, 1H, =C*H*), 7.32 (dt, *J* =5.0, 1.0 Hz, 1H, H5), 7.18–7.16 (m, 1H, H3), 7.03 (dd, *J* =5.0, 3.6 Hz, 1H, H4), 3.94 (s, 3H, OCH_3).

Isomer 2 (*Z*): [6]

^{1}H NMR (300 MHz, $CDCl_3$): δ [ppm] = 7.64 (s, 1H, =C*H*), 7.53 (dd, *J* = 5.2, 1.2 Hz, 1H, H5), 7.35 (dd, *J* = 3.7, 1.2 Hz, 1H, H3), 7.08 (dd, *J* = 5.2, 3.7 Hz, 1H, H4), 4.09 (s, 3H, OCH_3).

Cyclohexane-1,2-dione-bis-*O*-methyldioxime (L34)

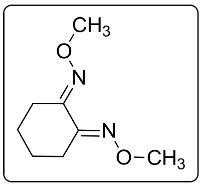

M [$C_8H_{14}N_2O_2$]: 170.21 g/mol.

Methoxyamine hydrochloride (500 mg, 5.90 mmol, 2.2 equiv) is suspended in anhydrous methanol (2 mL). Anhydrous pyridine (2 mL) is added and cyclohexane-1,2-dione (0.3 mL, 300 mg, 2.68 mmol, 2.2 equiv) is added dropwise. The reaction mixture is stirred for 8 h and another portion of methoxyamine hydrochloride (250 mg) is added. After complete conversion of starting material, the reaction mixture is taken up in dichloromethane and is washed with 5% citric acid aqueous solution (3 × 50 mL). The combined organic phases are

dried over $MgSO_4$ and filtered, and the solvent is evaporated in vacuo. The liquid residue is purified by flash column chromatography on silica gel (cyclohexane/EtOAc = 15:1). The product (439 mg, 2.58 mmol, 96%) is obtained as a colorless liquid.

***R*f**: 0.48 (cyclohexane/EtOAc = 4:1).

^{1}H NMR (300 MHz, $CDCl_3$): δ [ppm] = 3.98 (s, 6H, OC*H*$_3$), 2.61–2.56 (m, 4H, C*H*$_2$-3,-6), 1.64–1.59 (m, 4H, C*H*$_2$-4, -5).

(*Z*,*Z*)-1,3-Diphenyl-propane-1,3-dione-bis-*O*-methyloxime (L39)

(*Z*,*E*)-1,3-Diphenyl-propane-1,3-dione-bis-*O*-methyloxime (L39a)

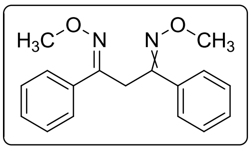

M [$C_{17}H_{18}N_2O_2$]: 282.34 g/mol.

Methoxyamine hydrochloride (410 mg, 4.91 mmol, 2.2 equiv) is suspended in anhydrous methanol (3 mL). Anhydrous pyridine is added, and dibenzoyl methane (500 mg, 2.23 mmol, 1.0 equiv) is added in small portions. The reaction mixture is stirred for 13 h at room temperature. After complete conversion the reaction mixture is taken up in dichloromethane and washed with 5% citric acid aqueous solution (3 × 50 mL). The combined organic phases are dried over $MgSO_4$ and filtered, and the solvent is evaporated in vacuo. The crude oily residue is purified by flash column on silica gel (cyclohexane/EtOAc=15:1). Isomer 1 (51 mg, 8%) and isomer 2 (15 mg, 2%) are obtained as colorless crystals. A mixed fraction (520 mg, 83%) is also obtained.

Total yield: 588 mg (2.08 mmol, 93%). (lit.: 83%) [7].

***R*f** (isomer 1): 0.28 (cyclohexane/EtOAc = 15:1).

R_f (Isomer 2): 0.22 (cyclohexane/EtOAc = 15:1).

mp: 57.3–58.2 °C (cyclohexane/EtOAc, mixture of isomers). (lit.: 58–59 °C (EtOH/H_2O, mixture of isomers) [7].

Isomer 1 (*Z*,*Z*):

^{1}H NMR (300 MHz, $CDCl_3$): δ [ppm] = 7.49–7.45 (m, 4H, H2, H6, H2', H6'), 7.31–7.25 (m, 6H, H3, H4, H5, H3', H4', H5'), 4.24 (s, 2H, CH_2-2), 3.97 (s, 6H, OCH_3).

Isomer 2 (*Z*,*E*):

^{1}H NMR (300 MHz, $CDCl_3$): δ [ppm] = 7.61–7.58 (m, 2H, H2, H6), 7.34–7.28 (m, 8H, H2', H3', H4', H5', H6', H3, H4, H5,), 4.02 (s, 2H, CH_2-2), 3.89 (s, 3H, OCH_3), 3.79 (s, 3H, OCH_3).

EA: Anal. calc.: 72.32% C; 6.43% H; 9.92% N; found: 71.86% C; 6.36% H; 9.93% N.

Salicylaldehyde-*O*-methyloxime (42)

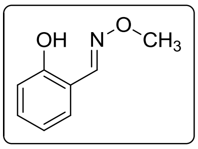

M [$C_8H_9NO_2$]: 151.16 g/mol.

Methoxyamine hydrochloride (376 mg, 4.50 mmol, 1.1 equiv) is suspended anhydrous methanol (3 mL). Anhydrous pyridine (3 mL) is added and salicyl aldehyde (0.43 mL, 500 mg, 4.09 mmol, 1.0 equiv) is added dropwise. The reaction mixture is stirred for 13 h at room temperature. The reaction mixture is taken up in dichloromethane and is washed with 5% citric acid aqueous solution (3 × 50 mL). The combined organic phases are dried over $MgSO_4$ and filtered, and the solvent is removed in vacuo. The oily residue is purified by flash

column chromatography on silica gel (cyclohexane/EtOAc = 15.1). The obtained oily product (583 mg, 3.86 mmol, 94%) crystallizes to a colorless solid.

***R*f**: 0.72 (cyclohexane/EtOAc = 4:1).

mp: ≤ 30 °C (cyclohexane/EtOAc). (lit.: 28–30 °C) [8].

^{1}H NMR (300 MHz, $CDCl_3$): δ [ppm] = 9.86 (s, 1H, O*H*), 8.16 (s, 1H, =C*H*), 7.29 (ddd, *J* = 7.3, 6.7, 1.7 Hz, 1H, H6), 7.15 (dd, *J* = 7.5, 1.7 Hz, 1H, H4), 7.01–6.97 (m, 1H, H5), 6.91 (td, *J* = 7.5, 1.1 Hz, 1H, H3), 3.99 (s, 1H, OCH_3).

2-Thiophene carbaldoxime (L37)

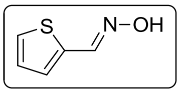

M [C_5H_5NOS]: 127.16 g/mol.

According to the general procedure of MacNevin [5] et al., hydroxylamine hydrochloride (1.86 g, 26.60 mmol, 1.5 equiv) is suspended in anhydrous ethanol (9 mL), and anhydrous pyridine (9 mL) is added. 2-Thiophene carbaldehyde (1.67 mL, 2.00 g, 17.83 mmol, 1.0 equiv) are added dropwise, and the reaction mixture is stirred for 48 h at room temperature. The solvent is removed in vacuo, and the liquid residue is taken up in water (25 mL) and is extracted with ethyl acetate (3 × 50 mL). The combined organic phases are dried over Na_2SO_4, and the solvent is removed in vacuo. The oily residue is dried in vacuo and the product (2.26 g, 17.77 mmol, quant., (lit.: quant.) [9]) crystallizes to a pale yellow solid.

***R*f**: 0.64 (cyclohexane/EtOAc = 1:1).

mp: 125–127.5 °C (EtOH). (lit.: 125-126 °C (EtOAc/*n*-hexane) [9]).

^{1}**H NMR** (300 MHz, MeOH-d$_4$): δ [ppm] = 7.71 (s, 1H, =C*H*), 7.62 (dt, *J* = 5.1, 1.1 Hz, 1H, H5), 7.43 (dd, *J* = 3.7, 1.1 Hz, 1H, H3), 7.10 (dd, *J* = 5.1, 3.7 Hz, 1H, H4).

Salicyl carbaldoxime (L41)

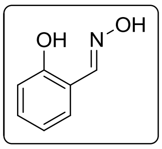

M [$C_7H_7NO_2$]: 137.14 g/mol.

Hydroxylamine hydrochloride (1.40 g, 20.00 mmol, 1.2 equiv) is suspended in anhydrous methanol (9 mL). Anhydrous pyridine (9 mL) is added, and salicyl aldehyde (1.71 mL, 2.00 g, 16.30 mmol, 1.0 equiv) is added dropwise. The reaction mixture is stirred for 48 h at room temperature. The solvent is removed in vacuo, and traces of pyridine are removed azeotropically with toluene (3 x 15 mL). The oily residue (2.45 g, 17.86 mmol, 90%) is dried in vacuo and crystallizes to pale yellow crystals, which can be recrystallized from *n*-hexane.

$\boldsymbol{R}_\mathbf{f}$: 0.48 (cyclohexane/EtOAc = 4:1).

mp: 57.5–58.0 °C (*n*-hexane). (lit.: 57 °C (benzene/petroleum ether) [10]).

^{1}**H NMR** (300 MHz, MeOH-d$_4$): δ [ppm] = 8.23 (s, 1H, =C*H*), 7.27–7.19 (m, 2H, H4, H6), 6.89–6.84 (m, 2H, H3, H5).

EA: Anal. calc.: 61.31% C; 5.14% H; 10.21% N; found: 61.04% C; 5.15% H; 10.40% N.

Cyclohexane-1,2-dione dioxime (L40)

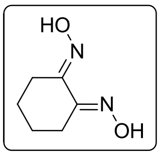

M [$C_6H_{10}N_2O_2$]: 142.16 g/mol.

According to a modified procedure of Hach [11] et al., hydroxylamine hydrochloride (780 mg, 11.10 mmol, 2.5 equiv) is dissolved in water (2 mL), and potassium hydroxide (620 mg, 11.10 mmol, 2.5 equiv), dissolved in water (2 mL), is added at 0 °C. Cyclohexane-1,2-dione (0.35 mL, 350 mg, 3.10 mmol, 1.0 equiv) is added dropwise at 0 °C. Subsequently, 15 mL of ethanol are added and the reaction mixture is stirred for 48 h at room temperature. The precipitate is filtered, and the filtrate is extracted with diethyl ether (3 × 50 mL). The combined organic phases are dried over Na_2SO_4, filtered and the solvent is removed in vacuo. The obtained solid is recrystallized from water and the product (380 mg, 2.67 mmol, 87%, (lit.: 74%) [11]) is obtained as colorless needles.

***R*f**: 0.12 (CH_2Cl_2/MeOH = 10:1).

mp: 183.2–184.6 °C, decomposition (H_2O). (lit.: 185–188 °C (H_2O) [11]).

^{1}H NMR (300 MHz, MeOH-d_4): δ [ppm] = 11.09 (s, 2H, O*H*), 2.44–2.42 (m, 4H, CH_2-4, -5), 1.49–1.48 (m, 4H, CH_2-3, -6).

EA: Anal. calc.: 50.69% C; 7.09% H; 19.71% N; found: 50.41% C; 7.15% H; 19.89% N.

2-*N*-(2-Thienylmethylene)aminobenzoic acid (L29)

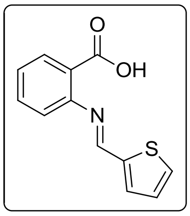

M [$C_{12}H_9NO_2S$]: 231.27 g/mol.

According to a procedure of Mohamed [12] et al., anthranilic acid (686 mg, 5.00 mmol, 1.0 equiv) is dissolved in ethanol (12 mL), 2-thiophene carbaldehyde (0.46 mL, 560 mg, 5.00 mmol, 1.0 equiv) and 750 mg of Na_2SO_4 are added, and the reaction mixture is heated for 13 h under reflux. The reaction mixture is filtered, and the filtrate is evaporated to dryness.

The obtained solid is recrystallized from ethanol and the product (546 mg, 2.36 mmol, 47%, (lit.: 80%) [12]) is obtained as yellow needles.

***R*$_f$**: 0.13 (cyclohexane/EtOAc = 4:1).

mp: 138.5–145.0 °C (EtOH). (lit.: 125 °C (EtOH) [12]).

^{1}H NMR (300 MHz, $CDCl_3$): δ [ppm] = 8.83 (s, 1H, =C*H*), 8.33 (dd, *J* = 7.8, 1.5 Hz, 1H, H6), 7.72–7.70 (m, 2H, H3, H5), 7.63–7.58 (m, 1H, H5‘), 7.45–7.29 (m, 2H, H3‘, H4‘), 7.24–7.20 (m, 1H, H4).

EA: Anal. calc.: 62.32% C; 3.92% H; 6.06% N; found: 62.06% C; 4.09% H; 6.04% N.

8-*N*-(2-Hydroxybenzylidene)aminoquinoline (L31)

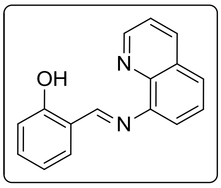

M [$C_{16}H_{12}N_2O$]: 248.28 g/mol.

8-Aminoquinoline (500 mg, 3.47 mmol, 1.0 equiv) is dissolved in anhydrous dichloromethane (5 mL), salicyl aldehyde (0.36 mL, 420 mg, 3.47 mmol, 1.0 equiv) and $MgSO_4$ (700 mg) are added. The reaction mixture is stirred for 13 h at room temperature. The reaction mixture is filtered, and the filtrate is washed with sat. $NaHCO_3$ solution (3 × 50 mL). The combined organic phases are dried over $MgSO_4$, and the solvent is removed in vacuo. The obtained red oil (0.70 g, 2.82 mmol, 81%) is dried in vacuo.

***R*$_f$**: 0.37 (cyclohexane/EtOAc = 4:1).

^{1}H NMR (300 MHz, $CDCl_3$): δ [ppm] = 13.95 (s, br, 1H, O*H*), 8.99 (dd, *J* = 4.2 Hz, 1.8 Hz, 1H, H2‘), 8.92 (s, 1H, =C*H*), 8.18 (dd, *J* = 8.2 Hz, 1.8 Hz, 1H, H4‘), 7.71 (dd, *J* = 8.2, 1.8 Hz,

1H, H7‘), 7.59–7.37 (m, 5H, H3‘, H5‘, H6‘, H3, H4), 7.10–7.07 (m, 1H, H6), 6.97–6.91 (m, 1H, H5).

Glyoxal-bis(*N*-phenylhydrazone) (L30)

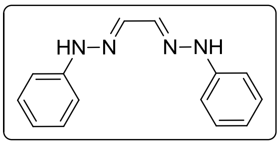

M [$C_{14}H_{14}N_4$]: 238.29 g/mol.

Glyoxal trimer dihydrate (350 mg, 5.00 mmol, 1.0 equiv) is suspended in water (0.4 mL), and phenyl hydrazine (0.98 mL, 1.08 g, 10.00 mmol, 2.0 equiv), dissolved in methanol (3 mL), is added dropwise. The reaction mixture is stirred for 13 h at room temperature. The orange precipitate (993 mg, 4.17 mmol, 83%, (lit.: 98%) [13]) is filtered, washed with ice cold water and dried in vacuo.

***R*$_\mathbf{f}$**: 0.47 (cyclohexane/EtOAc = 4:1).

mp: 165.7–167.5 °C (H_2O). (lit.: 167–168 °C) [13].

^{1}H NMR (300 MHz, DMSO-d_6): δ [ppm] = 10.36 (s, 2H, =C*H*), 7.63 (s, 2H, N*H*), 7.19 (t, *J* = 7.7 Hz, 4H, H3, H5, H3‘, H5‘), 6.97 (d, *J* = 7.7 Hz, 4H, H2, H6, H2‘, H6‘), 6.73 (t, *J* = 7.7 Hz, 2H, H4, H4‘).

EA: Anal. calc.: 70.57% C; 5.92% H; 23.51% N; found: 70.16% C; 5.92% H; 23.72% N.

***N*,*N*'-Bis(2-hydroxybenzylidene)-1,2-ethylenediamine (L32)**

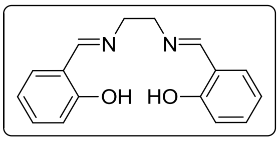

M [$C_{16}H_{16}N_2O_2$]: 268.31 g/mol.

According to a procedure of Zhao [14] et al., ethylene diamine (0.56 mL, 0.50 g, 8.32 mmol, 1.0 equiv) is dissolved in ethanol (10 mL), and salicyl aldehyde (1.75 mL, 2.03 g, 16.64 mmol, 2.0 equiv) is added dropwise at 0 °C. The reaction mixture is stirred for 13 h at room temperature. The yellow precipitate (2.23 g, 8.32 mmol, quant., (lit.: 97%) [14]) is filtered, washed with cold water and dried in vacuo.

***R*f**: 0.31 (cyclohexane/EtOAc = 4:1).

mp: 126.3–126.9 °C (H_2O). (lit.: 126 °C (EtOH) [15]).

^{1}H NMR (300 MHz, $CDCl_3$): δ [ppm] = 13.21 (s, br, 2H, O*H*), 8.36 (s, 2H, =C*H*), 7.32–7.21 (m, 4H, H4, H6, H4’, H6’), 6.96–6.93 (m, 2H, H5, H5’), 6.86 (td, *J* = 7.5, 1.1 Hz, 2H, H3, H3’), 3.94 (s, 4H, CH_2).

EA: Anal. calc.: 71.62% C; 6.01% H; 10.44% N; found: 71.30% C; 6.08% H; 10.28% N.

***N*,*N*’-Bis(2-thienylidene)-1,2-ethylene diamine (L28)**

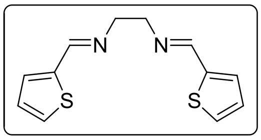

M [$C_{12}H_{12}N_2S_2$]: 248.37 g/mol.

According to a procedure of Zhao [14] et al., ethylene diamine (0.56 mL, 0.50 g, 8.32 mmol, 1.0 equiv) is dissolved in ethanol (10 mL) and 2-thiophene carbaldehyde (1.56 mL, 1.87 g, 16.64 mmol, 2.0 equiv) is added dropwise at 0 °C. The reaction mixture is stirred for 48 h at room temperature. The pale yellow precipitate (2.06 g, 8.32 mmol, quant., (lit.: 80%) [16]) is filtered, washed with cold water and dried in vacuo.

***R*f**: 0.53 (cyclohexane/EtOAc = 4:1).

mp: 90.0–91.0 °C (H_2O). (lit.: 91–93 °C (*n*-hexane) [16]).

^{1}**H NMR** (300 MHz, $CDCl_3$): δ [ppm] = 8.34 (s, 2H, =C*H*), 7.36 (dt, *J* = 5.0, 1.0 Hz, 2H, H5, H5‘), 7.25 (m, 2H, H3, H3‘), 7.03 (dd, *J* = 5.0, 3.6 Hz, 2H, H4, H4‘), 3.90 (s, 4H, CH_2).

EA: Anal. calcd for: 58.03% C; 4.87% H; 11.28% N; 25.82% S; found: 57.85% C; 4.68% H; 11.38% N; 26.08% S.

***N,N'*-Bis(2-pyridylmethylene)-1,2-ethylene diamine (L33)**

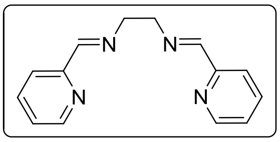

M [$C_{14}H_{14}N_4$]: 238.29 g/mol.

According to a modified procedure of Kouznetsov [17] et al., ethylene diamine (0.34 mL, 0.30 g, 5.00 mmol, 1.0 equiv) is dissolved in ethanol (6 mL) and 2-pyridine carbaldehyde (0.95 ml, 1.07 g, 10.00 mmol, 2.0 equiv) is added. The reaction mixture is heated under reflux for 15 h. The solvent is removed in vacuo and the oily residue crystallizes upon drying in vacuo. The dark red product is recrystallized from *n*-hexane and filtered and the precipitate is triturated with diethyl ether resulting in orange crystals (0.70 g, 2.94 mmol, 94%, (lit.: 86%) [17]).

$\boldsymbol{R_f}$: 0.35 (cyclohexane/EtOAc = 4:1).

mp: 62.0–63.2 °C (Et_2O). (lit.: 64.0–65.5 °C (petroleum ether) [18]).

^{1}**H NMR** (300 MHz, $CDCl_3$): δ [ppm] = 8.61–8.59 (m, 2H, H6, H6‘), 8.40 (s, 2H, =C*H*), 7.96 (d, *J* = 7.9 Hz, 2H, H3, H3‘), 7.70 (td, *J* = 7.9, 1.7 Hz, 2H, H4, H4‘), 7.30–7.25 (m, 2H, H5, H5‘), 4.04 (s, 4H, CH_2).

EA: Anal. calc.: 70.57% C; 5.92% H; 23.51% N; found: 70.30% C; 5.98% H; 23.94% N.

3-(*N*,*N*-Dimethylamino)propanoic acid hydrochloride (L3)

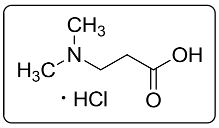

M [$C_5H_{12}ClNO_2$]: 153.61 g/mol.

According to a procedure of Clarke [19] and Rahal [20] et al., 3-aminopropanoic acid (3.56 g, 40.00 mmol, 1.0 equiv) is placed in a flask, and formic acid (99%, 25 mL) and aqueous formaldehyde solution (37%, 5 mL) are added. The reaction mixture is heated under reflux until evolution of CO_2 ceases (8–10 h). To the reaction mixture is added conc. hydrochloric acid (5 mL), and the mixture is evaporated to dryness. Traces of formic acid are removed azeotropically with toluene in vacuo (3 × 10 mL). The crude off-white solid is recrystallized from ethanol/acetone (20:1), and the product (3.38 g, 22.00 mmol, 55%, (lit.: 38%) [21]) is obtained as colorless crystals.

mp: 185.2–186.2 °C (EtOH/acetone). (lit.: 183–186 °C (2-propanol) [22]).

^{1}H NMR (300 MHz, MeOH-d$_4$): δ [ppm] = 3.42 (t, *J* = 6.9 Hz, 2H, NCH_2), 2.92 (s, 6H, CH_3), 2.87 (t, *J* = 6.9 Hz, 2H, α-CH_2).

***N*,*N*-Dimethyl-2-methylalanine hydrochloride (L5)**

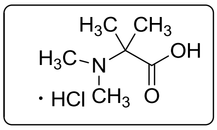

M [$C_6H_{14}ClNO_2$]: 167.63 g/mol.

According to a procedure of Clarke [19] and Rahal [20] et al., 2-methylalanine (4.12 g, 40.00 mmol, 1.0 equiv) is placed in the flask and formic acid (99%, 25 mL) and aqueous formaldehyde solution (37%, 5 mL) are added. The reaction mixture is heated under reflux

until evolution of CO_2 ceases (8–10 h). To the reaction mixture are added conc. hydrochloric acid (5 mL) and the mixture is evaporated to dryness. Traces of formic acid are removed azeotropically with toluene in vacuo (3 × 10 mL). The crude off-white solid is recrystallized from ethanol/acetone (20:1) and the product (5.60 g, 33.41 mmol, 84%, (lit.: 80%) [19]) is obtained as colorless crystals.

mp: 260.5–261.5 °C (EtOH/acetone). (lit.: 264 °C (HOAc) [19]).

^{1}H NMR (300 MHz, D_2O): δ [ppm] = 2.80 (s, 6H, N(C*H*$_3$)$_2$), 1.53 (s, 6H, C*H*$_3$).

EA: Anal. calc.: 42.99% C; 8.42% H; 8.36% N; found: 42.62% C; 8.49% H; 8.41% N.

(8-Quinolinyloxy)acetic acid hydrochloride (L11)

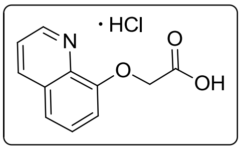

M [$C_{11}H_{10}ClNO_3$]: 239.66 g/mol.

According to a modified procedure of Neffe [23] et al., 8-hydroxyquinoline (530 mg, 3.63 mmol, 1.0 equiv) is suspended in hot ethanol (7 mL) and is heated under reflux. Potassium hydroxide (440 mg, 7.87 mmol, 2.2 equiv), dissolved in ethanol (6 mL), is added dropwise. Then, bromoacetic acid (520 mg, 3.73 mmol, 1.0 equiv) is added. The reaction mixture is cooled and is stirred for 15 h at room temperature. The precipitated solid is filtered and washed with cold ethanol. The colorless solid is dissolved in a few drops of water, and conc. hydrochloric acid is added dropwise. The precipitate is filtered and washed with cold ethanol and diethyl ether. The product (0.64 g, 2.67 mmol, 74%) is obtained as a yellow solid.

***R*$_f$**: 0.15 (DCM/MeOH = 10:1).

mp: 217.5–218.0 °C (EtOH) decomposition. (lit.: 216 °C (EtOH) [24]).

^{1}H NMR (300 MHz, D_2O): δ [ppm] = 8.94 (ddd, *J* = 8.5, 5.5, 1.5 Hz, 2H, H2, H4), 7.97 (dd, *J* =8.5, 5.5 Hz, 1H, H7), 7.71–7.65 (m, 2H, H3, H5), 7.38 (m, 1H, H6), 4.84 (s, 1H, CH_2).

1-Piperidinoacetic acid sodium salt (L15)

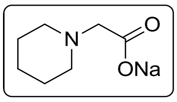

M [$C_7H_{12}NaNO_2$]: 165.17 g/mol.

According to a modified procedure of Lai [25] et al., bromoacetic acid (826 mg, 5.95 mmol, 1.0 equiv) is dissolved in water (4 mL), and of 3.3 M NaOH solution (2.5 mL) are added (pH=14). The mixture is cooled to 0 °C and piperidine (0.58 mL, 510 mg, 5.95 mmol, 1.0 equiv) are added dropwise. The reaction mixture is warmed to room temperature and is stirred for 72 h. The solvent is removed in vacuo and the crude piperidinoacetic acid sodium salt (1.56 g crude product, 61% w/w (0.94 g, 5.69 mmol, 95%) is obtained as a colorless solid.
mp: 217.5–218.0 °C (H_2O).
^{1}H NMR (300 MHz, D_2O): δ [ppm] = 3.21 (s, 2H, α-CH_2), 2.75 (t, *J* = 5.5 Hz, 4H, CH_2-2, -6), 1.66 (m, 4H, CH_2-3,-5), 1.50 (m, 2H, CH_2-4).

1-Pyrrolidinoacetic acid sodium salt (L9)

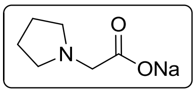

M [$C_6H_{10}NNaO_2$]: 151.14 g/mol.

According to a modified procedure of Lai [25] et al., bromoacetic acid (840 mg, 5.99 mmol, 1.0 equiv) is dissolved in water (4 mL), and 3.3 M NaOH solution (2.5 ml) is added (pH=14). The mixture is cooled to 0 °C, and pyrrolidine (0.49 mL, 0.42 g, 5.99 mmol, 1.0 equiv) is added dropwise. The reaction mixture is warmed to room temperature and is stirred for 72 h.

The solvent is removed in vacuo, and the obtained crude product (1.60 g, 51% w/w, 0.81g, 5.33 mmol, 89%) is dried in vacuo.

mp: decomposition at 255 °C.

^{1}H NMR (300 MHz, D_2O): δ [ppm] = 3.13 (s, 2H, α-CH_2), 2.61–2.56 (m, 4 H, CH_2-2, -5), 1.78–1.73 (m, 4H, CH_2-3, -4).

S-(2-Pyridyl)thioglycolic acid (L10))

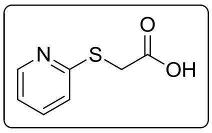

M [$C_7H_7NO_2S$]: 169.20 g/mol.

According to a modified procedure of Crosby [26] et al., 2-mercaptopyridine (500 mg, 4.50 mmol, 1.0 equiv) is dissolved in dichloromethane (10 mL) and triethylamine (1.60 mL, 1.10 g, 11.30 mmol, 2.5 equiv) is added. Bromoacetic acid (630 mg, 4.50 mmol, 1.0 equiv), dissolved in dichloromethane (3 mL), is added dropwise at 0 °C. The reaction mixture is warmed to room temperature and is stirred for 48 h. To the reaction mixture is added 2 M HCl solution until the aqueous phase is neutral, and then it is extracted with ethyl acetate (3 × 50 mL). The combined organic phases are dried over Na_2SO_4, and the solvent is removed in vacuo. The product (661 mg, 7.40 mmol, 87%) is obtained as an orange solid.

R$_f$: 0.14 (cyclohexane/EtOAc = 4:1).

mp: 124.0–133.2 °C decomposition (EtOAc). (lit.: 127–133 °C decomposition (H_2O) [27]).

^{1}H NMR (300 MHz, DMSO-d_6): δ [ppm] = 8.40 (ddd, 1H, *J* = 4.9, 1.8, 0.9 Hz, H6), 7.64 (ddd, 1H, *J* = 8.0, 7.4, 1.9 Hz, H4), 7.32 (dt, 1H, *J* = 8.1, 0.9 Hz, H3), 7.11 (ddd, 1H, *J* = 7.3, 4.9, 0.9 Hz, H5), 3.94 (s, 2H, CH_2).

Phenacyl-(2-pyridyl)thioether (L56)

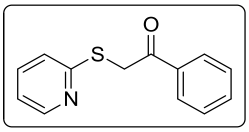

M [$C_{13}H_{11}NOS$]: 229.30 g/mol.

According to a modified procedure of Tang [28] et al., 2-mercaptopyridine (500 mg, 4.50 mmol, 1.0 equiv) is dissolved in anhydrous dichloromethane (6 mL) and triethylamine (0.94 mL, 680 mg, 6.00 mmol, 1.5 equiv) is added. Phenacyl bromide (900 mg, 4.50 mmol, 1.0 equiv), dissolved in anhydrous dichloromethane (2 mL), is added dropwise at 0°C. The reaction mixture is stirred for 72 h at room temperature. Sat. $NaHCO_3$ solution is added to the reaction mixture, and then it is extracted with ethyl acetate (3 x 30 mL). The combined organic phases are dried over Na_2SO_4 and filtered, and the solvent is removed in vacuo. The obtained brown oily residue (919 mg, 4.01 mmol, 89%, (lit.: 94%) [28]) is dried in vacuo.

R

$\boldsymbol{R_f}$: 0.47 (cyclohexane/EtOAc = 4:1).

^{1}H NMR (300 MHz, $CDCl_3$): δ [ppm] = 8.33 (ddd, *J* = 5.0, 1.9, 1.0 Hz, 1H, H6), 8.07–8.04 (m, 2H, H2’, H6’), 7.58–7.55 (m, 1H, H4), 7.50–7.44 (m, 3H, H3’, H4’, H5’), 7.25 (dt, *J* = 8.1, 1.0 Hz, 1H, H3), 6.97 (ddd, *J* = 7.3, 5.0, 1.1 Hz, 1H, H5), 4.70 (s, 2H, C*H*$_2$).

2-Picolinic acid methyl ester (2)

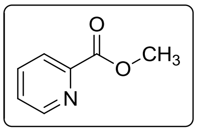

M [$C_7H_7NO_2$]: 137.14 g/mol.

According to a procedure of Baati [29] et al., picolinic acid (2.00 g, 16.25 mmol, 1.0 equiv) is dissolved in methanol (40 mL) and conc. sulfuric acid (1 mL) is added. The reaction mixture is heated under reflux for 48 h. The reaction mixture is neutralized with solid potassium

carbonate (adjusted to pH 8–9), and then it is extracted with ethyl acetate (3 × 50 mL). The combined organic phases are dried over Na_2SO_4, and the solvent is removed in vacuo. The crude product (1.92 g, 14.00 mmol, 86%, (lit.: 80%) [29]) is obtained as a pale yellow oil and is sufficiently pure for further use.

***R*$_f$**: 0.22 (cyclohexane/EtOAc = 4:1).

^{1}H NMR (300 MHz, $CDCl_3$): δ [ppm] = 8.72 (ddd, *J* = 4.7, 1.8, 1.0 Hz, 1H, H6), 8.11 (dt, *J* = 7.7, 1.0 Hz, 1H, H3), 7.82 (td, *J* = 7.7, 1.8 Hz, 1H, H4), 7.46 (ddd, *J* = 7.7, 4.7, 1.0 Hz, 1H, H5), 3-98 (s, 3H, COOCH_3).

2-Picolinehydroxamic acid (L13)

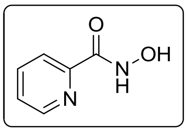

M [$C_6H_6N_2O_2$]: 138.12 g/mol.

According to a modified procedure of Tsukamoto [30] et al., hydroxylamine hydrochloride (1.01 g, 14.58 mmol, 2.0 equiv) and potassium hydroxide (1.23 g, 21.87 mmol, 3.0 equiv) are dissolved in water (10 mL). Picolinic acid methyl ester (1.00 g, 7.29 mmol, 1.0 equiv), dissolved in methanol (10 mL), is added dropwise at 0 °C. The reaction mixture is stirred for 24 h at room temperature. The reaction mixture is adjusted to pH 3–4 with 6 M hydrochloric acid, and the solvent is removed in vacuo. The warm solid residue is taken up in water until it dissolves, and it then crystallizes as colorless needles (570 mg, 4.13 mmol, 57%) upon cooling.

***R*$_f$**: 0.43 (DCM/MeOH = 10:1).

mp: 115.5–116.5 °C (H_2O). (lit.: 117–118 °C ($CHCl_3$) [31]).

^{1}**H NMR** (300 MHz, MeOH-d_4): δ [ppm] = 8.59 (ddd, *J* = 4.8, 1.7, 1.1 Hz, 1H, H6), 8.04 (dt, *J* = 7.7, 1.1 Hz, 1H, H3), 7.95 (td, *J* =7.7, 1.7 Hz, 1H, H4), 7.53 (ddd, *J* = 7.7, 4.8, 1.1 Hz, 1H, H5).

***N*,*N*-Dimethyl-8-aminoquinoline (L43)**

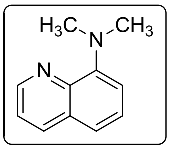

M [$C_{11}H_{12}N_2$]: 172.23 g/mol.

According to a modified procedure of Chen [32] et al., 8-aminoquinoline (0.43 g, 3.00 mmol, 1.0 equiv) is dissolved in acetonitrile (10 mL), water (2 mL) and aqueous formaldehyde solution (2.40 mL, 37%) are added. At 0 °C sodium cyanoborohydride (0.57 g, 9.00 mmol, 3.0 equiv) is added in small portions, and acetic acid (0.6 mL) is added dropwise. The reaction mixture is stirred for 15 h at room temperature. To the reaction mixture is added saturated citric acid solution (10 mL) and it is stirred for 48 h. The mixture is adjusted to pH 10 with 4 M NaOH solution and it is extracted with *n*-hexane (3 x 30 mL). The combined organic phases are washed with water (1 x 30 mL), dried over Na_2SO_4 and filtered, and the solvent is removed in vacuo. The crude product (0.48 g, 2.79 mmol, 93%) is obtained as a yellow oil and is sufficiently pure for further use.

$\boldsymbol{R}_\mathbf{f}$: 0.47 (cyclohexane/EtOAc = 4:1).

^{1}**H NMR** (300 MHz, $CDCl_3$): δ [ppm] = 8.89 (dd, *J* = 4.1, 1.8 Hz, H2), 8.09 (dd, 1H, *J* = 8.3, 1.8 Hz, H4), 7.45–7.34 (m, 3H, H3, H5, H7), 7.12 (dd, *J* = 7.1, 1.8 Hz, 1H, H6), 3.09 (s, 6H, CH_3).

2-(2-Methylbutanoyl)cyclohexanone (L50)

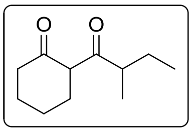

M [$C_{11}H_{18}O_2$]: 182.26 g/mol.

According to a procedure of Hünig [33] et al., 1-(pyrrolidino)-1-cyclohexene (1.60 mL, 1.50 g, 10.00 mmol, 1.0 equiv) and triethylamine (1.52 ml, 1.11 g, 11.00 mmol, 1.1 equiv) are dissolved in anhydrous toluene (25 mL). 2-Methylbutyric acid chloride (1.24 mL, 1.20 g, 10.00 mmol, 1.0 equiv) is added dropwise. The reaction mixture is stirred for 13 h at room temperature. To the reaction mixture is added 20% hydrochloric acid (10 mL) and it is heated under reflux for 2 h. It is extracted with toluene (3 × 50 mL) and the aqueous phase is adjusted to pH 5–6 with 4 M NaOH solution and extracted with toluene. The combined organic phases are washed with water (1 x 30 mL), dried over Na_2SO_4 and filtered, and the solvent is removed in vacuo. The crude oily residue is purified by flash column chromatography on silica gel (cyclohexane/EtOAc= 30:1). The product (530 mg, 2.91 mmol, 29%) is obtained as a pale yellow oil.

***R*f**: 0.64 (cyclohexane/EtOAc = 8:1).

^{1}H NMR (300 MHz, $CDCl_3$): δ [ppm] = 2.67 (m, 1H, α-C*H*), 2.40–2.30 (m, 4H, CH_2-3, CH_2-6), 1.76–1.61 (m, 6H, CH_2-4, CH_2-5, CH_2CH$_3$), 1.38 (m, 1H, C*H*-2‘), 1.07 (d, J = 6.8 Hz, 3H,CH_3), 0.88 (t, J = 7.5 Hz, 3H, CH$_2$CH_3).

^{13}C NMR (75.5 MHz, $CDCl_3$): δ [ppm] = 204.4 (1‘-C=O), 183.8 (1-C=O), 106.3 (α-*C*H), 39.9 (*C*H-2‘), 31.6 (C-6), 26.5 (C-3), 23.7 (C-5), 23.0 (C-4), 21.6 (*C*H$_2$CH$_3$), 16.6 (*C*H$_3$), 11.9 (CH$_2$*C*H$_3$).

IR (ATR): ν = 2987, 2935, 2872, 1723, 1699, 1587, 1449, 1414, 1214 cm^{-1}.

HPLC-MS: *m/z (%)* = 183.0 (100) $[M+H]^+$, 184.1 (16) $[M+2H]^+$, 205.0 (24) $[M+Na]^+$. Method A, t_R= 3.65 min.

ESI-HRMS: *m/z (%)* = calc. for $[C_{11}H_{18}O_2+Na]^+$: 205.1204, found.: 205.1199.

_N_-Methylanthranilic acid (L4)

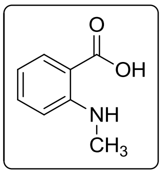

M $[C_8H_9NO_2]$: 151.16 g/mol.

According to a procedure of Liu [34] et al., anthranilic acid (1.37 g, 10.00 mmol, 1.0 equiv) is dissolved in ethanol (15 mL), and dimethyl sulfate (3.80 mL, 5.04 g, 40 mmol, 4.0 equiv) is added dropwise. The reaction mixture is heated under reflux for 48 h. Sodium hydroxide (4 g, 0.10 mol, 10.0 equiv), dissolved in water (20 mL), is added and the reaction mixture is heated under reflux for further 2 h. After cooling, the solvent is removed in vacuo, and the residue is adjusted to pH 6–7. The obtained pale orange crystals (570 mg, 3.77 mmol, 38%) are filtered and dried in vacuo.

***R*f**: 0.27 (cyclohexane/EtOAc = 4:1).

mp: 170.0–171.6 °C (H_2O). (lit.: 170–173 °C) [35].

^{1}H NMR (300 MHz, DMSO-d_6): δ [ppm] = 7.77 (dd, *J* = 7.9, 1.6, 1H, H6), 7.37 (ddd, *J* = 8.5, 7.3, 1.6 Hz, 1H, H4), 6.67 (d, *J* = 8.5, 1 H, H5), 6.55 (t, *J* = 7.3 Hz, 1H, H3), 2.82 (s, 3H, NCH_3).

EA: Anal. calc.: 39.10% C; 7.87% H; 9.12% N; found: 38.82% C; 7.91% H; 9.11% N.

DL-*S*-Methylcysteine (L8)

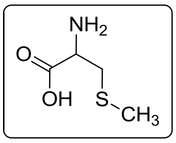

M [$C_4H_9NO_2S$]: 135.18 g/mol.

According to a procedure of Wlostowski [36] et al., L-cysteine (611 mg, 5.00 mmol, 1.0 equiv) is dissolved in methanol (7 mL) and *N,N,N‘,N‘*-tetramethylguanidine (1.32 mL, 1.21 g, 10.50 mmol, 2.1 equiv) is added. Methyl iodide (0.31 mL, 712 mg, 5.00 mmol, 1.0 equiv) is added dropwise and the reaction mixture is stirred for 1 h at 50 °C. After being cooled, the solvent is evaporated in vacuo, and to the liquid residue is added an equimolar amount of conc. HCl (0.4 mL conc. HCl in 1 mL methanol). The precipitate is filtered and recrystallized from ethanol. The product (0.47 g, 3.48 mmol, 70%, (lit.: 93%) [36]) is obtained as a colorless solid.

$\boldsymbol{R_f}$: 0.12 (cyclohexane/EtOAc = 4:1). Staining with ninhydrin TLC reagent.

mp: 216.9–219.2 °C decomposition (EtOH). (lit.: 219–220 °C [36]).

^{1}H NMR (300 MHz, D_2O): δ [ppm] = 3.90 (dd, *J* = 7.6, 4.3 Hz, 1H, α-C*H*), 3.01 (ddd, 2H, *J* = 15.0, 6.0, 4.3 Hz, CH_2), 2.11 (s, 3H, SCH_3).

EA: Anal. calc.: 35.54% C; 6.71% H; 10.36% N; 23.72% S; found: 35.24% C; 7.07% H; 10.53% N; 23.17% S.

DL-*N*-Acetyl-*S*-methylcysteine (L12)

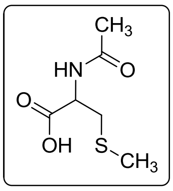

M [$C_6H_{11}NO_3S$]: 177.22 g/mol.

According to a procedure of Terayama [37] et al., DL-*S*-methylcysteine (100 mg, 0.74 mmol, 1.0 equiv) is dissolved in 1 M NaOH solution, and acetic acid anhydride (76 µL, 83 mg, 0.81 mmol, 1.1 equiv) is added dropwise at 0 °C. After the addition, the reaction mixture is warmed to room temperature and is stirred for 2 h. The reaction mixture is adjusted to pH 1 with 6 M hydrochloric acid and extracted with ethyl acetate (5 x 5 mL). The combined organic phases are dried over $MgSO_4$ and filtered, and the solvent is removed in vacuo. The oily residue crystallizes to an off-white solid (86 mg, 0.49 mmol, 66%, (lit. 81%) [37]) upon drying in vacuo.

***R*$_f$**: 0.61 (cyclohexane/EtOAc = 4:1). Staining with iodine.

mp: 145.0–145.7 °C (EtOAc). (lit.: 147–149 °C (EtOH) [38]).

^{1}H NMR (300 MHz, D_2O): δ [ppm] = 4.57 (dd, 1H, *J* = 8.2, 4.8 Hz, α-C*H*), 2.95 (ddd, 2H, *J* = 22.4, 14.1, 6.5 Hz, CH_2), 2.11 (s, 3H, SCH_3), 2.02 (s, 3H, (C=O)CH_3).

Sarcosine methylester hydrochloride (3)

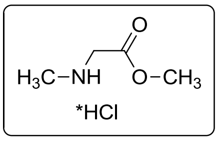

M [$C_4H_{10}ClNO_2$]: 139.58 g/mol.

According to a procedure of O'Connell [39] et al., sarcosine (2.50 g, 28.00 mmol, 1.0 equiv) is suspended in anhydrous methanol (50 mL) and it is cooled to 0 °C. Thionyl chloride

(2.14 mL, 3.51 g, 29.46 mmol, 1.05 equiv) is added dropwise, it is stirred for 30 min at 0 °C, and then the mixture is heated under reflux for 3 h. The solvent is removed in vacuo. The solid residue is dissolved in methanol (10 mL), diethyl ether (40 mL) is added, and the solution is cooled to 0 °C. The precipitate is filtered and dried in vacuo. The product (3.60 g, 25.79 mmol, 92%, (lit.: 90%) [39]) is obtained as a colorless solid.

R

$\boldsymbol{R_f}$: 0.16 (cyclohexane/EtOAc = 1:2, 1% NEt_3). Staining with vanilline/sulfuric acid TLC reagent.

mp: 116.1–117.9 °C (MeOH/Et_2O). lLit.: 117–119 °C (MeOH/Et_2O) [40]).

^{1}H NMR (300 MHz, MeOH-d_4): δ [ppm] = 3.99 (s, 2H, C*H*$_2$), 3.84 (s, 3H, COOC*H*$_3$), 2.77 (s, 3H, NC*H*$_3$).

2-Hydroxymethyl thiophene (4)

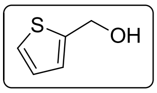

M [C_5H_6OS]: 114.17 g/mol.

2-Thiophene carbaldehyde (0.83 mL, 2.00 g, 17.84 mmol, 1.0 equiv) is dissolved in anhydrous ethanol (10 mL), and sodium borohydride (1.01 g, 26.76 mmol, 1.5 equiv) is added portionwise so that the temperature does not raise above 40 °C. After complete conversion the reaction mixture is quenched with water (30 mL) and extracted with dichloromethane (3 × 50 mL). The combined organic phases are dried over Na_2SO_4 and the solvent is removed in vacuo. The obtained pale yellow and oily residue (1.70 g, 14.89 mmol, 84%) is sufficiently pure for further use.

$\boldsymbol{R_f}$: 0.27 (cyclohexane/EtOAc = 4:1).

^{1}H NMR (300 MHz, $CDCl_3$): δ [ppm] = 7.27 (dd, *J* = 4.9, 1.4 Hz, 1H, H5), 7.02–6.96 (m, 2H, H4, H3), 4.80 (s, 2H, C*H*$_2$).

2-Bromomethyl thiophene (5)

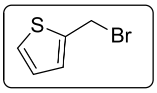

M [C_5H_5BrS]: 177.06 g/mol.

According to a procedure of Huynh [41] et al., 2-hydroxymethyl thiophene (909 mg, 7.96 mmol, 1.0 equiv) is dissolved in anhydrous diethyl ether (15 mL) and is cooled to 0 °C. Phosphorous tribromide (0.75 mL, 2.15 g, 7.96 mmol, 1.equiv) is added dropwise at 0 °C. The reaction mixture is stirred for 1.5 h at 0°C and is warmed then to room temperature. After complete conversion, water is added cautiously (30 mL), and the mixture is extracted with diethyl ether (3 × 50 mL). The combined organic phases are dried over Na_2SO_4 and filtered, and the solvent is removed in vacuo. The crude product (1.32 g, 7.46 mmol, 93%, (lit.: 89%) [41]) is obtained as pale yellow oil and is sufficiently pure for further use.

***R*$_f$**: 0.75 (cyclohexane/EtOAc = 4:1).

^{1}H NMR (300 MHz, $CDCl_3$): δ [ppm] = 7.33 (dd, *J* = 5.1, 1.2 Hz, 1H, Ar-H5), 7.13 (ddt, *J* = 3.5, 1.2, 0.7 Hz, 1 H, H4), 6.95 (dd, *J* = 5.1, 3.5 Hz, 1H, H3), 4.77 (s, 2H, C*H*$_2$).

***N*-(2-Thienylmethyl)sarcosine methylester (6)**

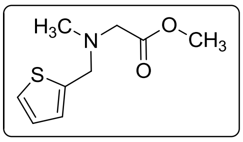

M [$C_9H_{13}NO_2S$]: 199.27 g/mol.

Sarcosine methylester hydrochloride (1.04 g, 7.43 mmol, 1.0 equiv) is suspended in anhydrous dichloromethane and triethylamine (3.1 mL, 2.26 g, 22.3 mmol, 3.0 equiv), and 2-bromomethyl thiophene (1.32 g, 7.43 mmol, 1.0 equiv) is added dropwise at 0 °C. The reaction mixture is stirred for 72 h at room temperature. Then, sat. $NaHCO_3$ solution is added

and the mixture is extracted with ethyl acetate (3 × 50 mL). The combined organic phases are dried over Na_2SO_4 and filtered, and the solvent is removed in vacuo. The liquid residue is purified by flash column chromatography on silica gel (cyclohexane:EtOAc = 4:1, 1% NEt_3). The product (306 mg (1.54 mmol, 21%) is obtained as a pale yellow oil.

***R*f**: 0.26 (cyclohexane/EtOAc = 4:1, 1% NEt_3).

^{1}H NMR (300 MHz, $CDCl_3$): δ [ppm] = 7.26–7.23 (m, 1H, H5), 6.95–6.91 (m, 2H, H3, H4), 3.92 (s, 2H, NC*H*2), 3.70 (s, 3H, COOC*H*3), 3.29 (s, 2H, α-C*H*2), 2.43 (s, 3H, NC*H*3).

^{13}C NMR (75.5 MHz, $CDCl_3$): δ [ppm] = 171.3 (C=O), 141.2 (C-2), 126.5 (C-4), 126.4 (C-3), 125.3 (C-5), 56.7 (α-*C*H2), 55.1 (N*C*H2), 51.5 (COO*C*H3), 42.0 (N*C*H3).

IR (ATR): ν = 3108, 3076, 2950, 2843, 1735, 1434, 1372, 1283, 1198, 1175, 1124, 1040, 971, 852, 755, 699.

HPLC-MS: *m/z (%)* = 97.1 (12) $[C_5H_5S]^+$, 116.0 (10) $[M-C_5H_5S]^+$, 199.9 (100) $[M+H]^+$, 200.9 (9) $[M+2H]^+$. method A. t_R= 0.2 min.

ESI-HRMS: *m/z (%)* = calcd for $[C_9H_{13}NO_2S+Na]^+$: 222.0565, found: 222.0578.

N-(2-Thienylmethyl)sarcosine sodium salt (L18)

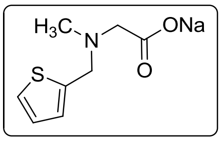

M [$C_8H_{10}NNaO_2S$]: 207.23 g/mol.

N-(2-Thienylmethyl)sarcosine methylester (101.5 mg, 0.55 mmol, 1.0 equiv) is dissolved in methanol (0.3 mL), THF (0.5 mL) is added and the solution is cooled to 0 °C. To the reaction mixture is added 4 M NaOH solution (0.5 mL), and the reaction mixture is stirred at room temperature until complete conversion is accomplished. The solvent is evaporated, and the product (113.7 mg, 0.55 mmol, quant.) is obtained as a pale yellow solid.

mp: decomposition at 350 °C.

^{1}**H NMR** (300 MHz, D_2O): δ [ppm] = 7.24 (dd, *J* = 4.9, 1.4 Hz, 1H, H5), 6.90–6.86 (m, 2H, H3, H4), 3.73 (s, 2H, NCH_2), 2.88 (s, 2H, α-CH_2), 2.13 (s, 3H, NCH_3).

^{13}**C NMR** (75.5 MHz, D_2O): δ [ppm] = 178.1 (C=O), 139.3 (C-2), 172.7 (C-4), 126.7 (C-3), 125.7 (C-5), 59.0 (α-CH_2), 53.5 (NCH_2), 40.9 (NCH_3).

IR (ATR): ν = 3392, 3104, 2949, 2793, 2493, 1866, 1775, 1596, 1420, 1254, 1201, 1124, 1026, 989, 878, 695.

ESI-MS: *m/z (%)* = 97.1 (12) $[C_5H_5S]^+$, 185.9 (100) $[M+H]^+$, 186.9 (20) $[M+2H]^+$, 207.9 (29) $[M+Na]^+$.

ESI-HRMS: *m/z (%)* = calc. for $[C_8H_{11}NO_2S+Na]^+$: 208.0408, found: 208.0405.

Diphenyl-(8-quinolinyloxy)phosphinate (L21)

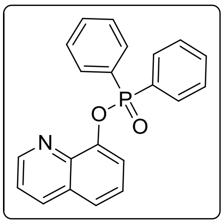

M $[C_{21}H_{16}NO_2P]$: 345.33 g/mol.

According to a modified procedure of Langer [42] et al., 8-hydroxyquinoline (2.00 g, 13.78 mmol, 1.0 equiv) is dissolved in anhydrous diethyl ether, and triethylamine (2.88 mL, 2.11 g, 20.68 mmol, 1.5 equiv) and diphenylphosphine chloride (2.53 mL, 3.04 g, 13.78 mmol, 1.0 equiv) are added dropwise at 0 °C. The reaction mixture is warmed to room temperature and stirred for 72 h at room temperature. To the mixture is added *n*-hexane, it is then filtered, and the filter cake is washed with diethyl ether. The filtrate is evaporated to dryness, and the solid residue is triturated with diethyl ether and filtered. The product (1.29 g, 3.92 mmol, 66%) is obtained as a colorless solid.

R_f: 0.34 (cyclohexane/EtOAc = 1:1).

mp: 112.2–113.7 °C (Et_2O).

^{1}H NMR, COSY, HSQC, HMBC (400 MHz, $CDCl_3$): δ [ppm] = 9.00 (dd, *J* = 4.2, 1.6 Hz, 1H, H2), 8.18–8.11 (m, 4H, H2‘, H6‘, H2‘‘, H6‘‘), 8.10 (dd, *J* = 8.4, 1.6 Hz, 1H, H4), 7.85 (dt, *J* = 7.7, 1.6 Hz, 1H, H7), 7.54 (d, *J* = 8.1 Hz, 1H, H5), 7.50–7.46 (m, 2H, H4‘, H4‘‘), 7.44–7.39 (m, 5H, H3, H3‘, H5‘, H3‘‘, H5‘‘), 7.36 (d, *J* = 8.1 Hz, 1H, H6).

^{13}C NMR, HSQC, HMBC (100.6 MHz, $CDCl_3$): δ [ppm] = 150.2 (C-2), 147.2 (d, J_{CP} = 8.4 Hz, C-8), 141.2 (d, J_{CP} = 5.0 Hz, C-8a), 135.8 (C-4), 132.4 (d, J_{CP} = 2.8 Hz, C-1‘, C-1‘‘), 132.3 (d, J_{CP} = 3.8 Hz, C-2‘, C-2‘‘, C-6‘, C-6‘‘), 132.3 (d, J_{CP} = 10.5 Hz, C-4’, C-4‘‘), 129.6 (C-4a), 128.6 (d, J_{CP} = 13.6 Hz, C-3‘, C-3‘‘, C-5‘, C-5‘‘), 126.4 (C-6), 124.1 (C-5), 121.7 (C-3), 119.9 (d, J_{CP} = 4.3 Hz, C-7).

^{31}P-NMR (MHz, $CDCl_3$): δ [ppm] = 32.1 (s).

IR (ATR): ν = 1617, 1497, 1469, 1386, 1313, 1225, 1129, 1110, 1087, 1057, 914, 819, 754, 728 cm^{-1}.

HPLC-MS: *m/z (%)* = 346.1 (100) $[M+H]^+$, 347.1 (24) $[M+2H]^+$. Method B, t_R= 1.85 min.

EA: Anal. calc.: 73.04% C; 4.67% H; 4.06% N; found: 72.78% C; 4.57% H; 4.07% N.

Dicyclohexyl-(8-quinolinoxy)phosphinite (L20)

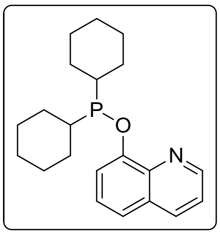

M [$C_{21}H_{28}NOP$]: 341.43 g/mol.

According to a modified procedure of Langer [42] et al., 8-hydroxyquinoline (94 mg, 0.64 mmol, 1.0 equiv) is dissolved in anhydrous THF (3 mL), and triethylamine (0.18 mL, 130 mg, 1.29 mmol, 2.0 equiv) is added under an argon atmosphere. Dicyclohexylphosphine

chloride (0.14 ml, 150 mg, 0.64 mmol, 1.0 equiv) is added dropwise at 0 °C. The reaction mixture is warmed to room temperature and is stirred for 8 h at room temperature. *n*-Pentane is added and the mixture is stored at −27 °C for 48 h to precipitate nonconverted 8-hydroxyquinoline. The supernatant is taken off and is evaporated in vacuo. The resulting pale yellow oily residue is directly used in the Ullmann model reactions.

^{1}H NMR (300 MHz, MeCN-d$_3$): δ [ppm] = 8.77 (dd, *J* = 4.2, 1.6 Hz, 1H, H2), 8.15 (dd, *J* = 8.3, 1.6 Hz, 1H, H4), 7.47–7.40 (m, 2H, H5, H7), 7.32 (dd, *J* = 8.3, 1.2 Hz, 1H, H3), 7.17 (dd, *J* = 7.5, 1.2 Hz, 1H, H6), 1.87–1.22 (m, 22H, C*H*$_2$-cyclohexyl).

Difuryl-(8-quinolinoxy)phosphinite (L19)

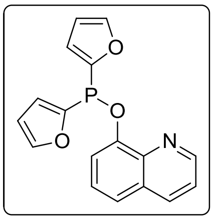

M [$C_{17}H_{12}NO_3P$]: 309.26 g/mol.

According to a modified procedure of Langer [42] et al., 8-hydroxyquinoline (109 mg, 0.75 mmol, 1.0 equiv) is dissolved in anhydrous THF (3 mL) and triethylamine (0.21 mL, 150 mg, 1.50 mmol, 2.0 equiv) is added under argon atmosphere. Difurylphosphine chloride (0.12 mL, 150 mg, 0.75 mmol, 1.0 equiv) is added dropwise at 0 °C. The reaction mixture is warmed to room temperature and is stirred for 2 h. *n*-Hexane is added and the supernatant is taken off and evaporated under an oil pump vacuum. The resulting violet oily residue is directly used in the Ullmann model reactions.

^{1}H NMR (300 MHz, MeCN-d$_3$): δ [ppm] = 8.95 (dd, *J* = 4.2, 1.5 Hz, 1H, H2), 8.38 (dd, *J* = 8.3, 1.5 Hz, H4), 7.57 (d, *J* = 8.0 Hz, 1H, H7), 7.43 (dd, *J* = 8.3, 4.2 Hz, 2H, H3, H5),

7.28-7.25 (m, 1H, H6), 7.21–7.19 (m, 2H, H, H5', H5''), 6.68 (dtd, J = 6.9, 3.6, 1.8 Hz, 4H, H3', H3'', H4', H4'').

Di-*tert.*-butyl-(8-quinolinoxy)phosphinite (L26)

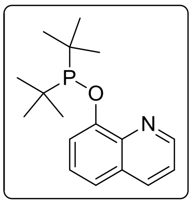

M [$C_{17}H_{24}NOP$]: 289.35 g/mol.

According to a modified procedure of Langer [42] et al., 8-hydroxyquinoline (121 mg, 0.83 mmol, 1.0 equiv) is dissolved in 3 mL anhydrous THF, and triethylamine (0.23 mL, 170 mg, 1.66 mmol, 2.0 equiv) is added under argon atmosphere. Di-*tert*-butylphosphine chloride (0.16 mL, 150 mg, 0.83 mmol, 1.0 equiv) is added dropwise at 0 °C. The reaction mixture is warmed to room temperature and is stirred for 2 h. *n*-Pentane is added and the supernatant is taken off and evaporated in vacuo. The resulting colorless solid is directly used in the Ullmann model reactions.

^{1}H NMR (300 MHz, MeCN-d_3): δ [ppm] = 8.92 (dd, 1H, J = 4.2, 1.6 Hz, H2), 8.37 (dd, J = 8.4, 1.6 Hz, 1H, H4), 7.63–7.55 (m, 2H, H5, H7), 7.50 (dd, J = 8.3, 1.4 Hz, H3), 7.2 (dd, 1H, J = 7.4, 1.4 Hz, H6), 1.30 (d, 18 H, J_{HP} = 12.2 Hz , C(CH_3)$_3$).

Diethyl-(8-quinolinoxy)phosphite (L27)

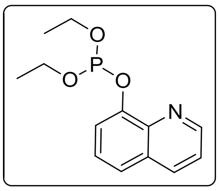

M [$C_{13}H_{16}NO_3P$]: 265.24 g/mol.

According to a modified procedure of Langer [42] et al., 8-hydroxyquinoline (121 mg, 0.83 mmol, 1.0 equiv) is dissolved in anhydrous THF (3 mL), and triethylamine (0.23 mL, 170 mg, 1.66 mmol, 2.0 equiv) is added under argon atmosphere. Diethylchlorophosphite (0.12 ml, 130 mg, 0.83 mmol, 1.0 equiv) is added dropwise at 0 °C. The reaction mixture is warmed to room temperature and stirred for 2 h. *n*-Pentane (30 mL) and anhydrous acetonitrile (10 mL) are added to the mixture, and the supernatant is taken off and evaporated in vacuo. The resulting yellow solid is directly used in the Ullmann model reactions.

^{1}H NMR (300 MHz, MeOH-d_4): δ [ppm] = 8.88 (dd, *J* = 4.6, 1.6 Hz, 1H, H2), 8.54 (dd, *J* = 8.3, 1.6 Hz, 1H, H4), 7.68 (m, *J* = 8.4, 4.6 Hz, 1H, H7), 7.54 (d, *J* = 7.2 Hz, 1H, H5), 7.50 (dd, *J* = 8.3, 1.7 Hz, 1H, H3), 7.24 (dd, *J* = 7.2, 1.7 Hz, 1H, H6), 3.21 (q, *J* = 7.3 Hz, 4H, CH_2CH_3), 1.32 (t, *J* = 7.3 Hz, 6H, CH_3).

Diphenyl-(2-Pyridyl)phosphinite (L25)

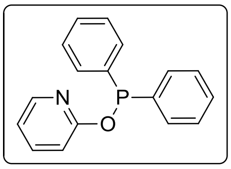

M [$C_{17}H_{14}NOP$]: 279.27 g/mol.

According to a modified procedure of Langer [42] et al., 2-hydroxypyridine (380 mg, 4.00 mmol, 1.0 equiv) is suspended in anhydrous THF (8 mL) and triethylamine (1.11 mL, 811 mg, 8.00 mmol, 2.0 equiv) is added under an argon atmosphere. Diphenylphosphine chloride (0.74 mL, 883 mg, 4.00 mmol, 1.0 equiv) is added dropwise at 0 °C. The reaction mixture is warmed to room temperature and is stirred for 72 h. *n*-Hexane is added, and the supernatant is taken off and evaporated in vacuo. The resulting yellow solid is directly used in the Ullmann model reactions.

^{1}H NMR (300 MHz, $CDCl_3$): δ [ppm] = 8.19 (ddd, J = 4.9, 2.0, 0.7 Hz, 1H, H6), 7.98–7.89 (m, 4H, H2‘, H2‘‘, H6‘, H6‘‘), 7.78–7.62 (m, 1H, H4), 7.55–7.42 (m, 6H, H3‘, H3‘‘, H4‘, H4‘‘, H5‘, H5‘‘), 7.19 (ddd, J = 8.2, 1.6, 0.8 Hz, 1H, H3), 7.06–6.98 (ddd, 1H, H5).

Diphenyl-(2-Hydroxymethylthienyl)phosphinite (L22)

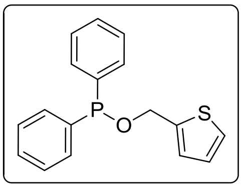

M [$C_{17}H_{15}OPS$]: 298.34 g/mol.

According to a modified procedure of Langer [42] et al., 2-hydroxymethyl thiophene (500 mg, 4.38 mmol, 1.0 equiv) is dissolved in 8 mL anhydrous THF, and triethylamine (1.21 mL, 894 mg, 8.76 mmol, 2.0 equiv) is added under an argon atmosphere. Diphenylphosphine chloride (0.89 mL, 1.06 g, 4.82 mmol, 1.1 equiv) is added dropwise at 0 °C. The reaction mixture is warmed to room temperature and is stirred for 72 h under argon atmosphere. *n*-Hexane is added, and the supernatant is taken off and evaporated in vacuo. The resulting yellow oil is directly used in the Ullmann model reactions.

^{1}H NMR (300 MHz, $CDCl_3$): δ [ppm] = 7.85–7.78 (m, 4H, H2‘, H2‘‘, H6‘, H6‘‘), 7.52–7.40 (m, 6H, H3‘, H3‘‘, H4‘, H4‘‘, H5‘, H5‘‘), 7.30 (dd, J = 5.1, 1.3 Hz, 1H, H5), 7.01–6.98 (m, 1H, H4), 6.93 (dd, J = 5.1, 3.5 Hz, 1H, H3), 5.23 (d, J_{HP} = 7.1, 0.7 Hz, 2H, CH_2).

General procedure for Ullmann model reactions of the ligand screening:

In a heat-dried reaction tube with a stirring bar (length 0.6 cm) is placed anhydrous potassium phosphate (430 mg, 2.00 mmol, 2.0 equiv) under an argon atmosphere in counterflow. Copper iodide (14 mg, 0.10 mmol, 10 mol %), ligand (0.10 mmol, 10 mol %), 4-methoxyphenol

(124 mg, 1.00 mmol, 1.0 equiv), 4-bromoanisole (130 μL, 187 mg, 1.00 mmol, 1.0 equiv) and anhydrous acetonitrile (1 mL) are placed in the reaction tube, and it is capped with a septum and furnished with an argon balloon. The reaction mixture is stirred for 30 min at room temperature and is then placed in a heating bath at 80 °C. Samples of 500 μL of the reaction mixture are taken after 2 h 15 min and 24 h. The samples are filtered over Celite and washed with dichloromethane, and the filtrate is evaporated in vacuo. The conversion of starting material is determined by ^{1}H NMR spectroscopy.

Spectra

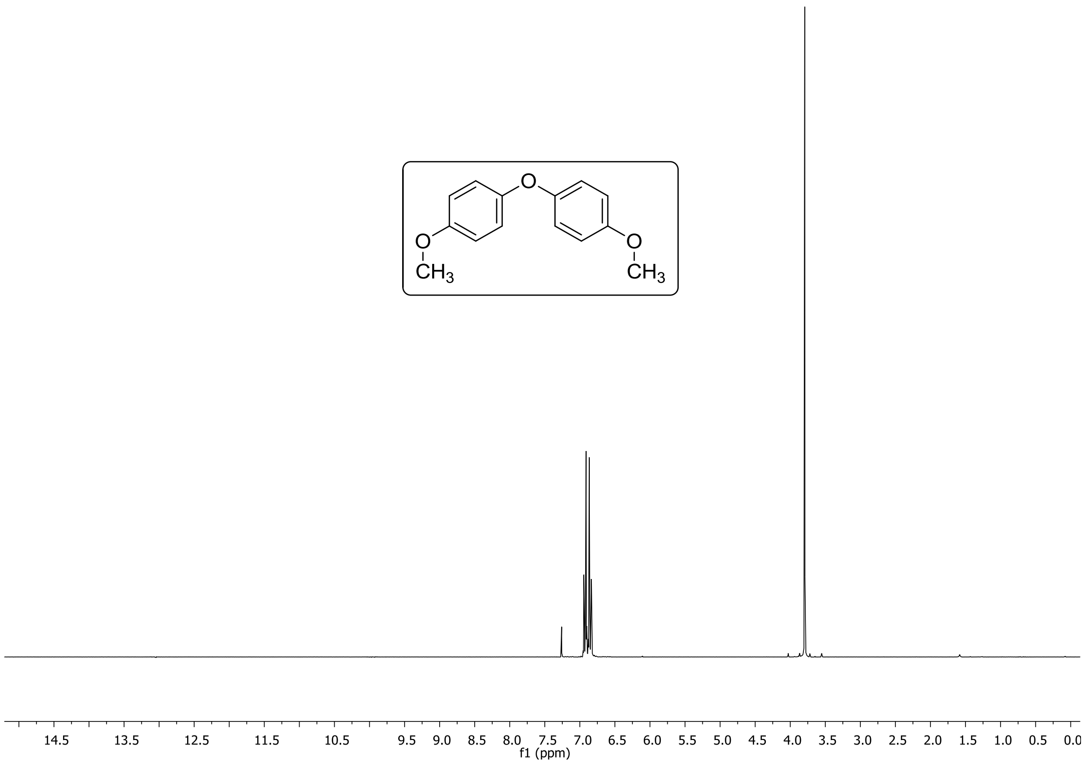

^{1}H NMR in $CDCl_3$ (300 MHz): **Compound 1**.

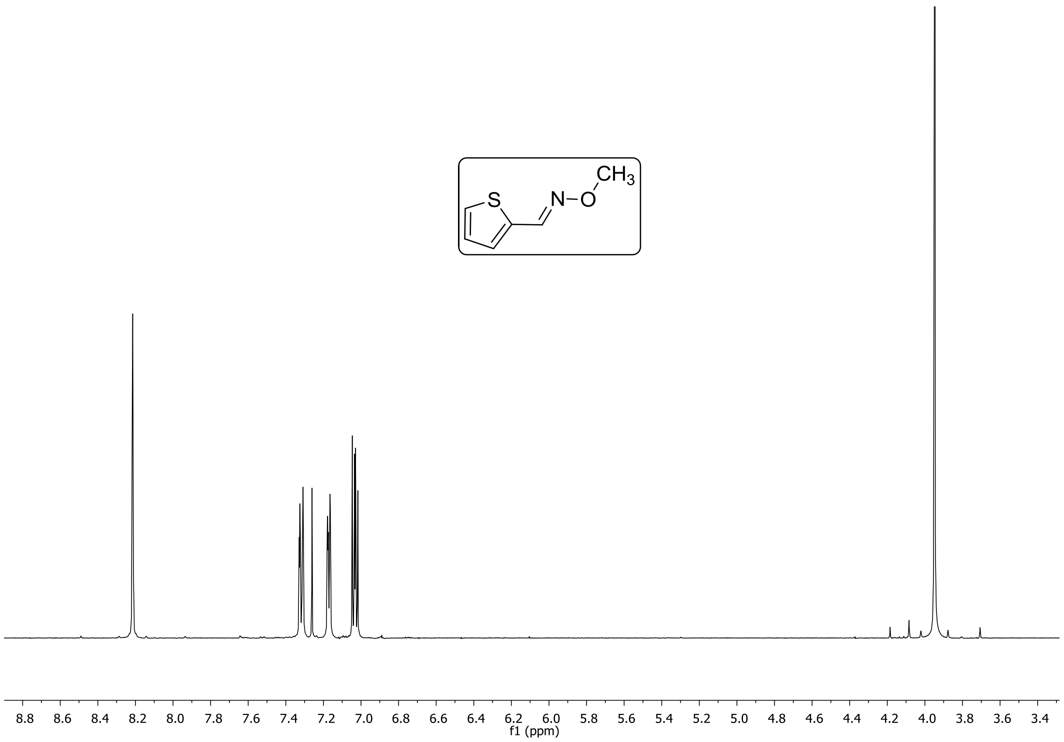

^{1}H NMR in $CDCl_3$ (300 MHz): **Compound L36a**.

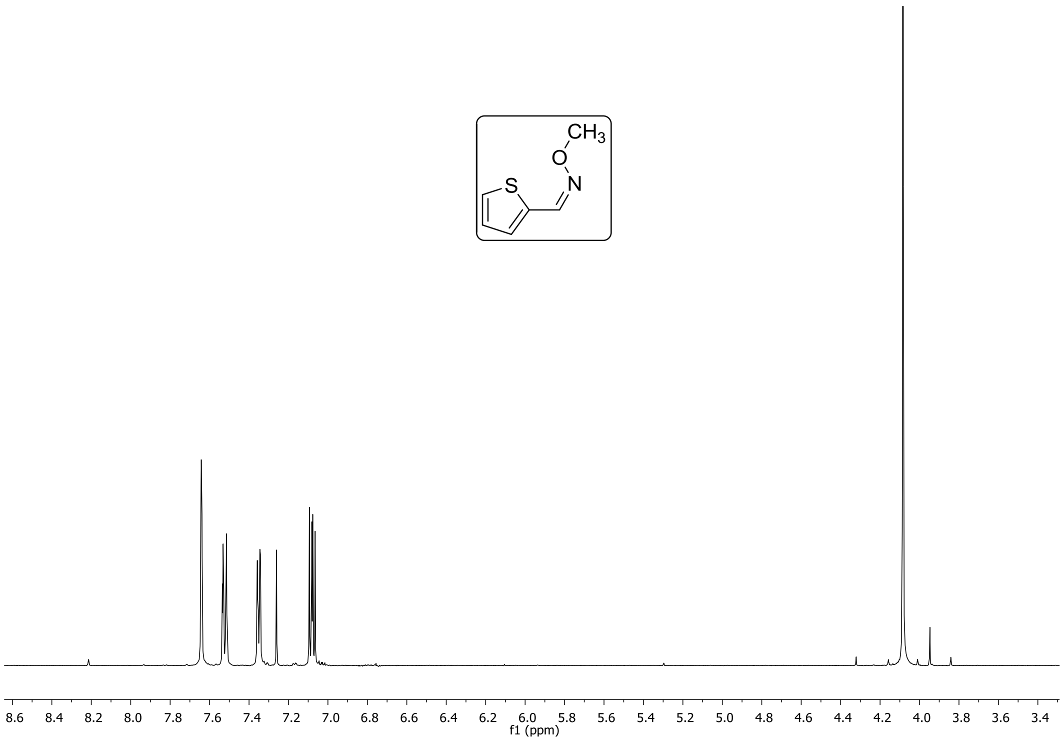

^{1}H NMR in $CDCl_3$ (300 MHz): **Compound L36**.

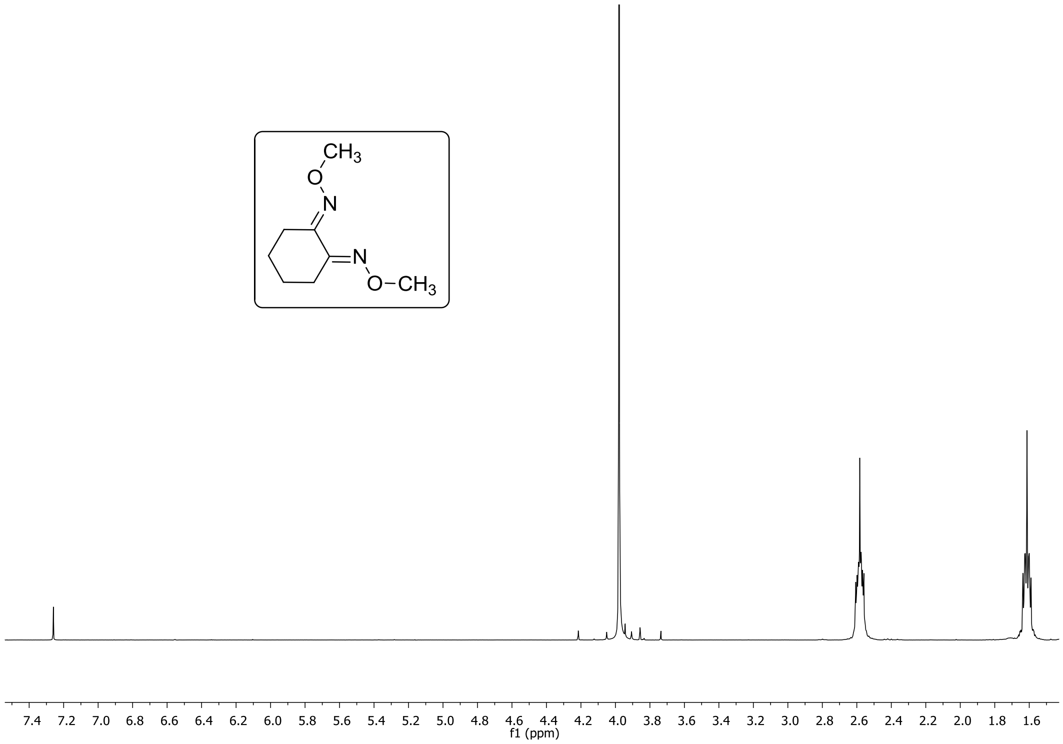

^{1}H NMR in $CDCl_3$ (300 MHz): **Compound L34**.

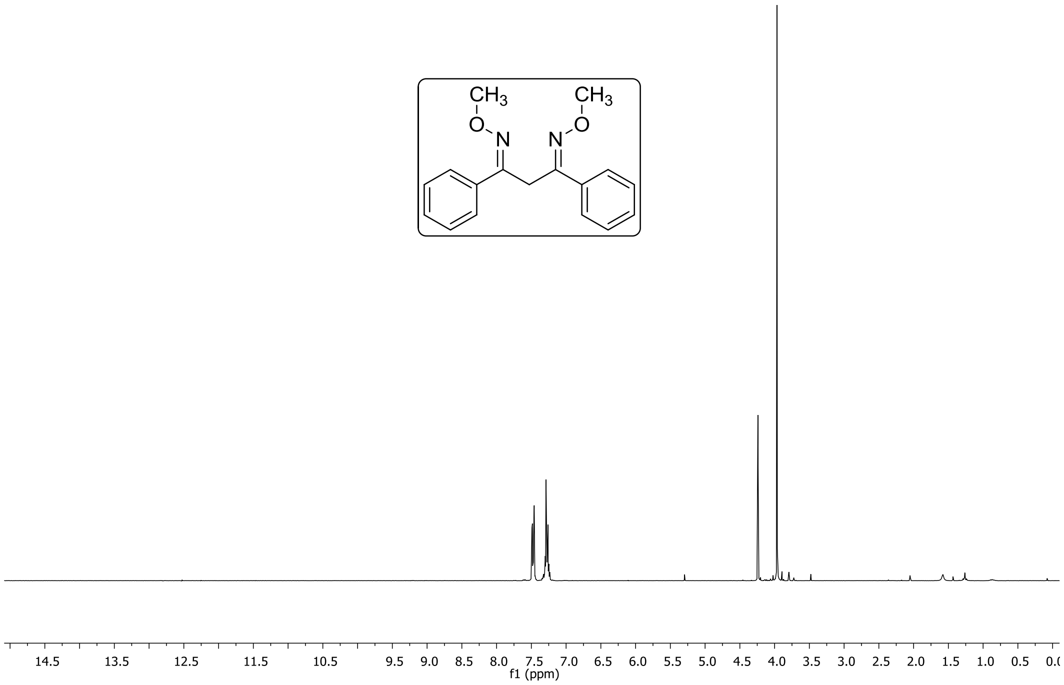

^{1}H NMR in $CDCl_3$ (300 MHz): **Compound L39**.

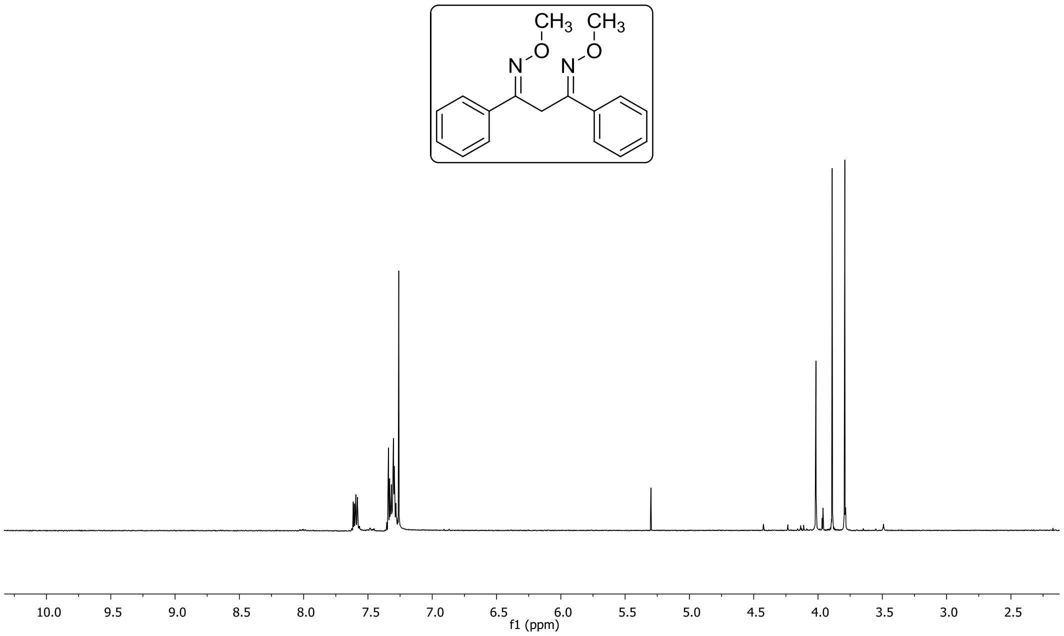

^{1}H NMR in $CDCl_3$ (300 MHz): **Compound L39a**.

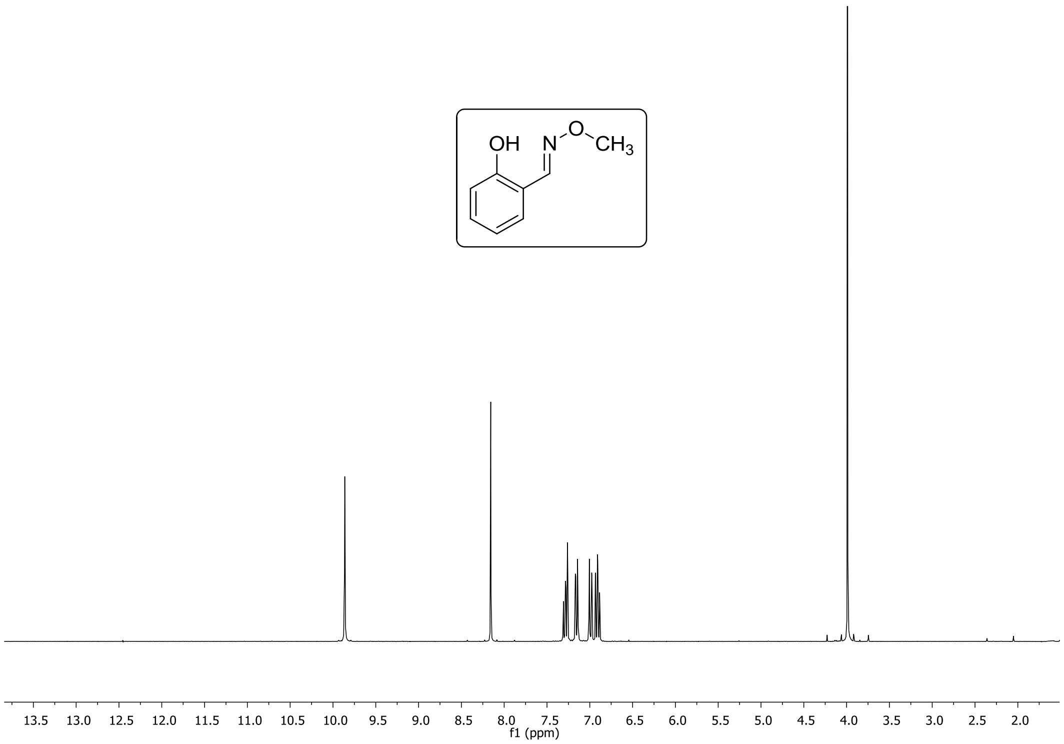

^{1}H NMR in $CDCl_3$ (300 MHz): **Compound L42**.

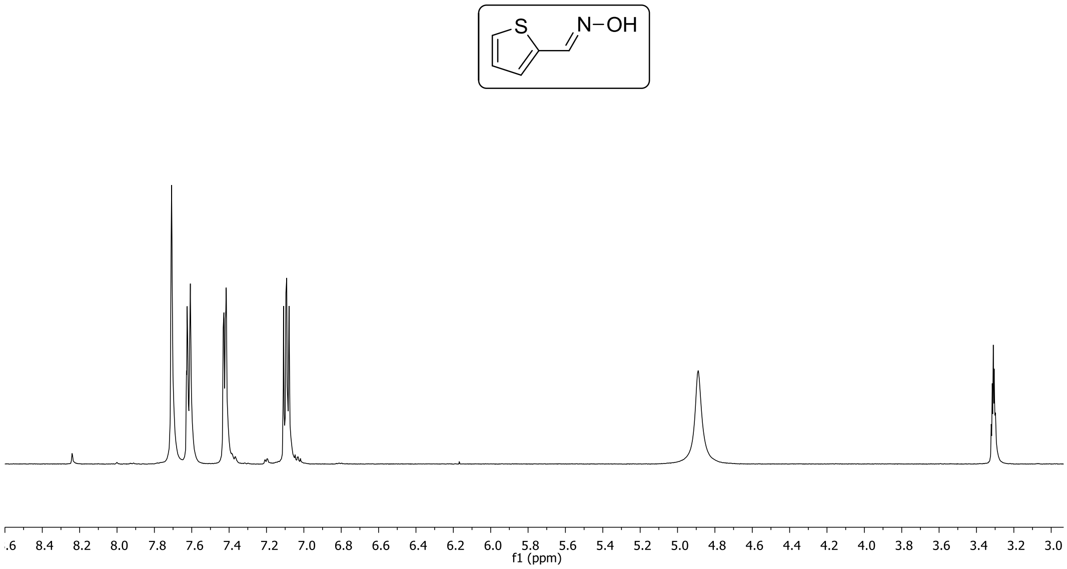

^{1}H NMR in MeOH-d_4 (300 MHz): **Compound L37**.

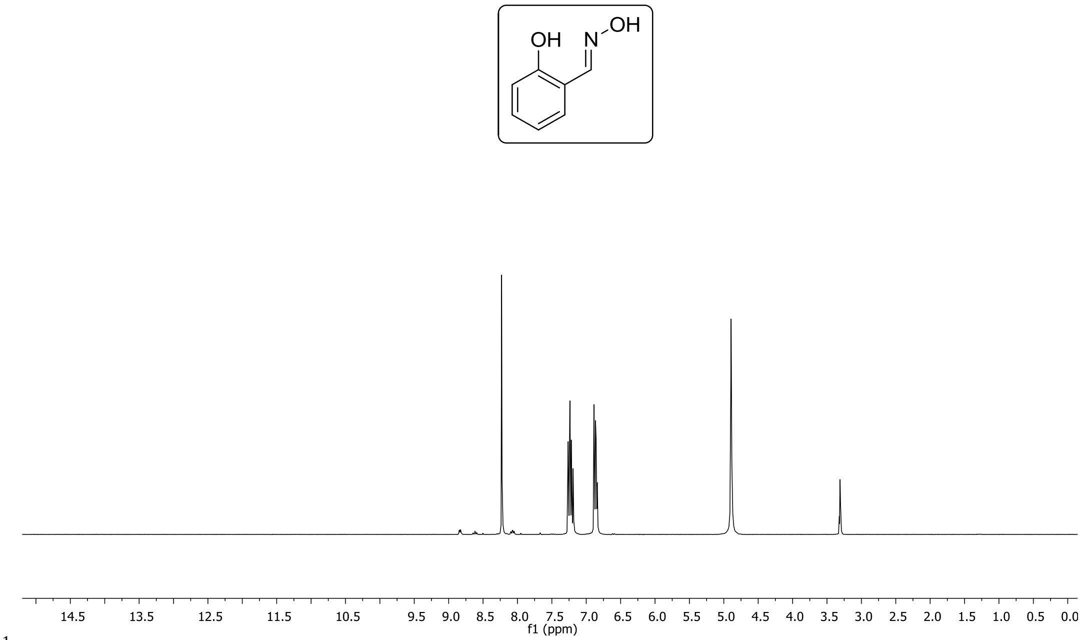

^{1}H NMR in MeOH-d_4 (300 MHz): **Compound L41**.

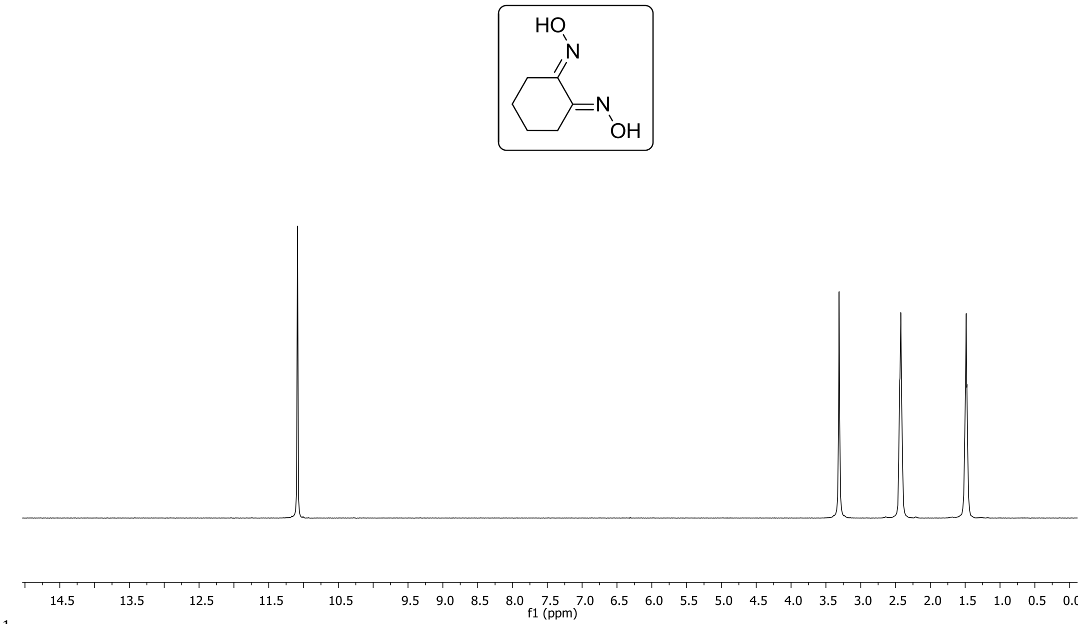

^{1}H NMR in MeOH-d_4 (300 MHz): **Compound L40**.

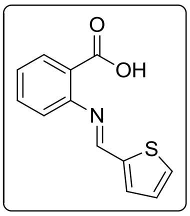

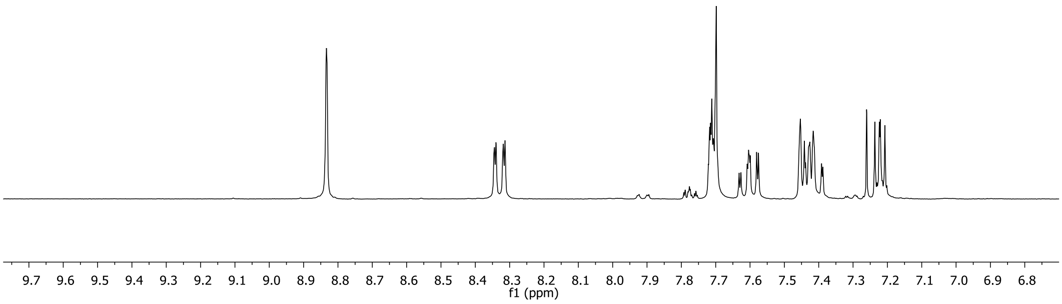

^{1}H NMR in $CDCl_3$(300 MHz): **Compound L29**.

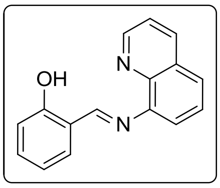

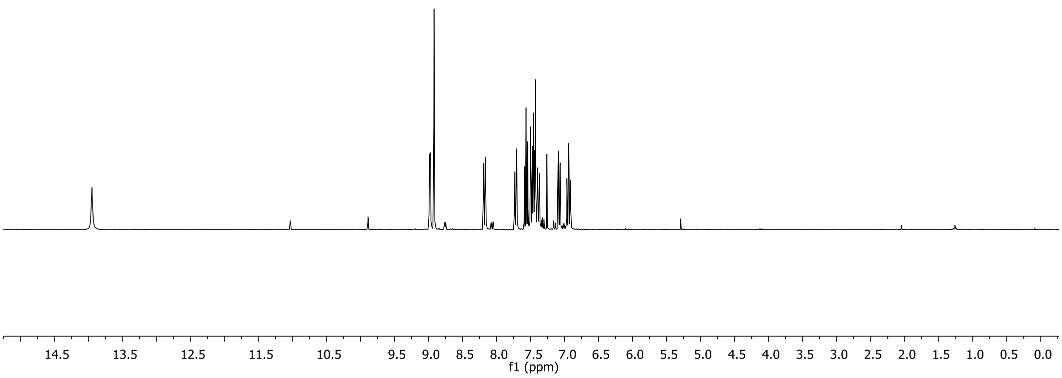

^{1}H NMR in $CDCl_3$(300 MHz): **Compound L31**.

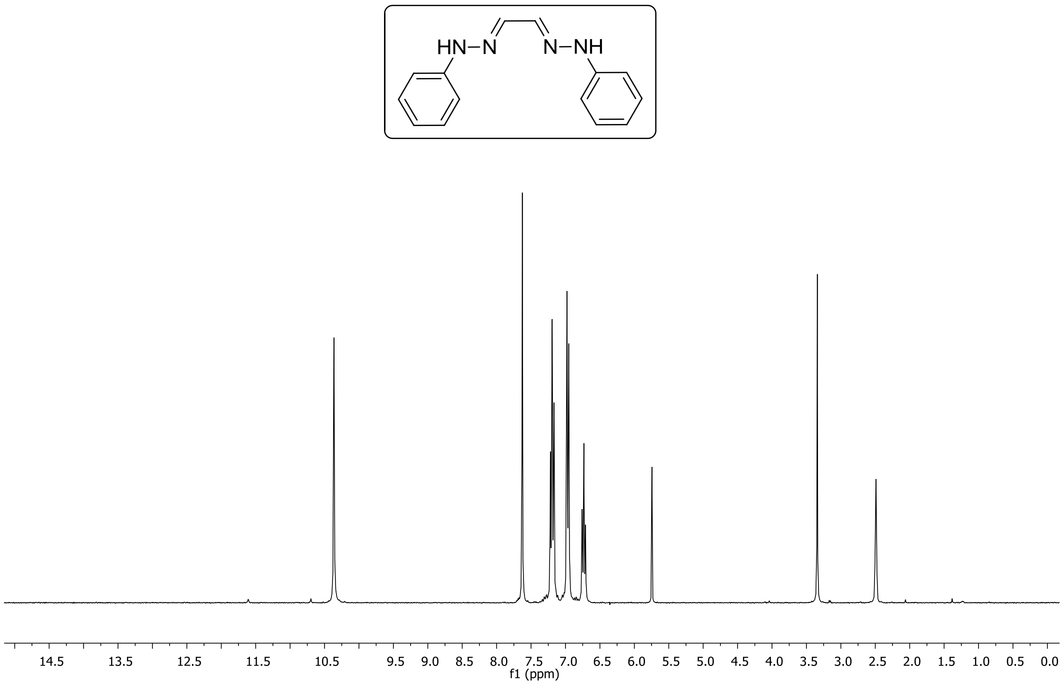

^{1}H NMR in DMSO-d_6 (300 MHz): **Compound L30**.

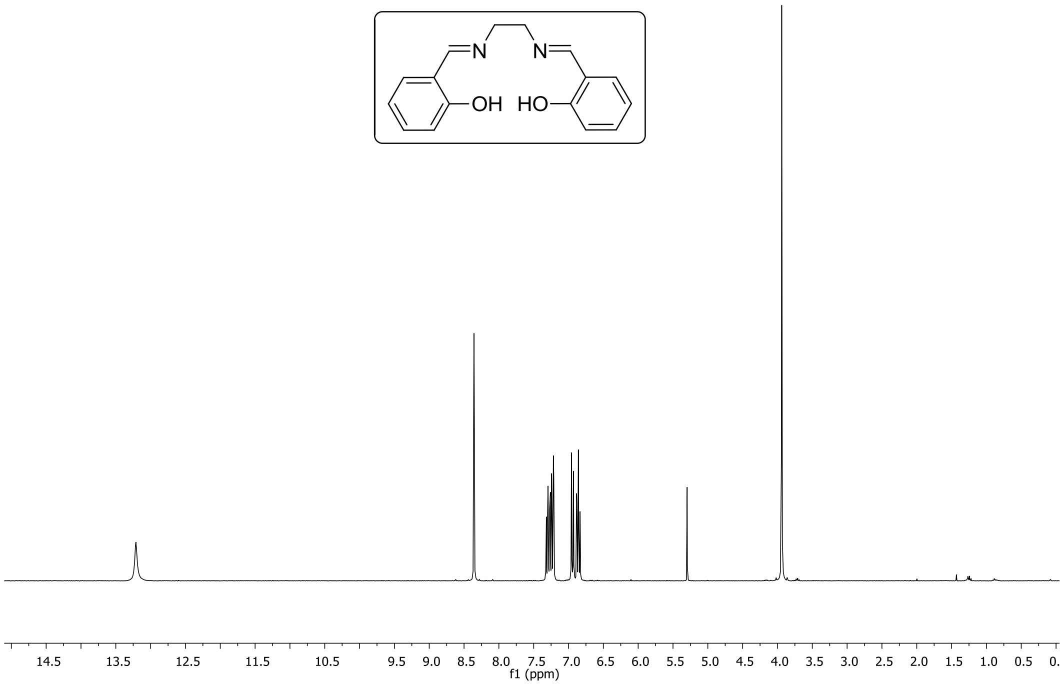

^{1}H NMR in $CDCl_3$ (300 MHz): **Compound L32**.

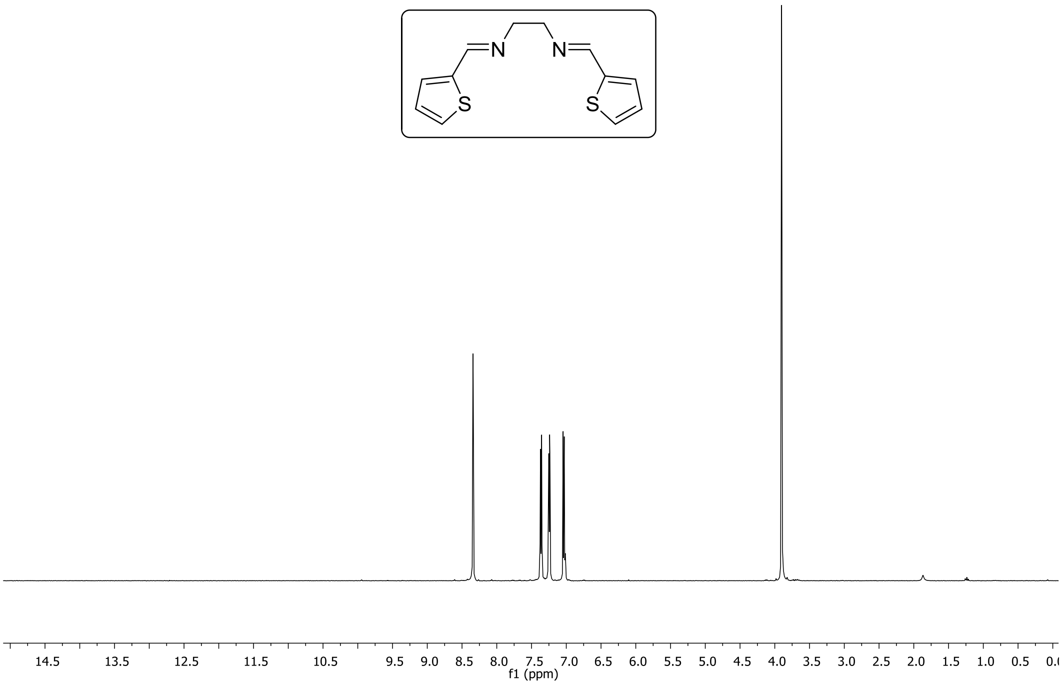

^{1}H NMR in $CDCl_3$ (300 MHz): **Compound L28**.

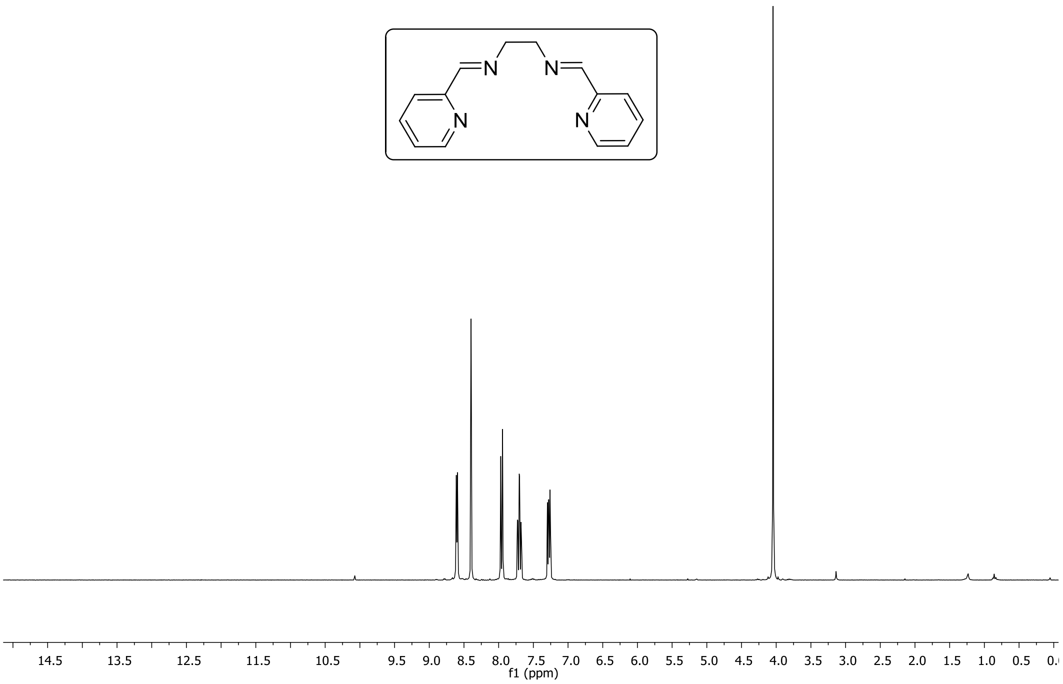

^{1}H NMR in $CDCl_3$ (300 MHz): **Compound L33**.

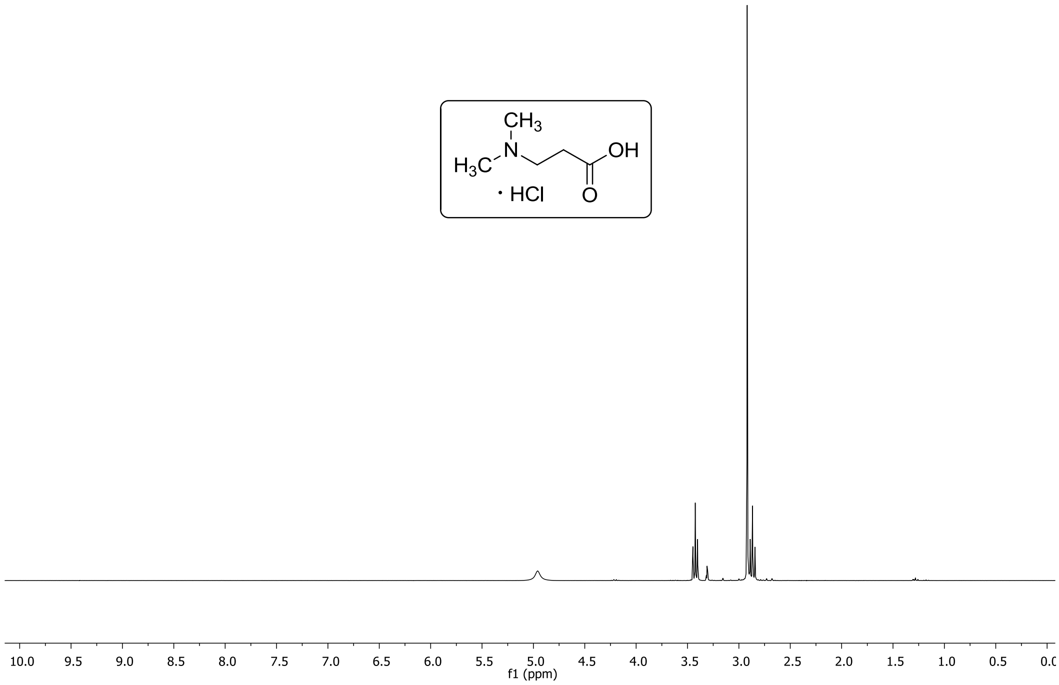

^{1}H NMR in MeOH-d_4 (300 MHz): **Compound L3.**

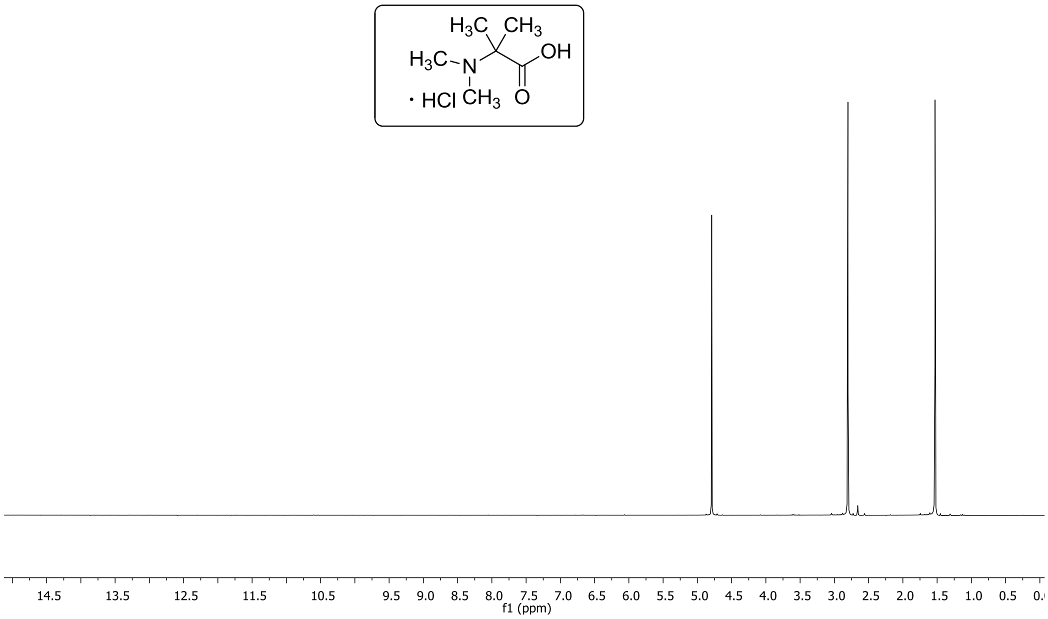

^{1}H NMR in D_2O (300 MHz): **Compound L5**.

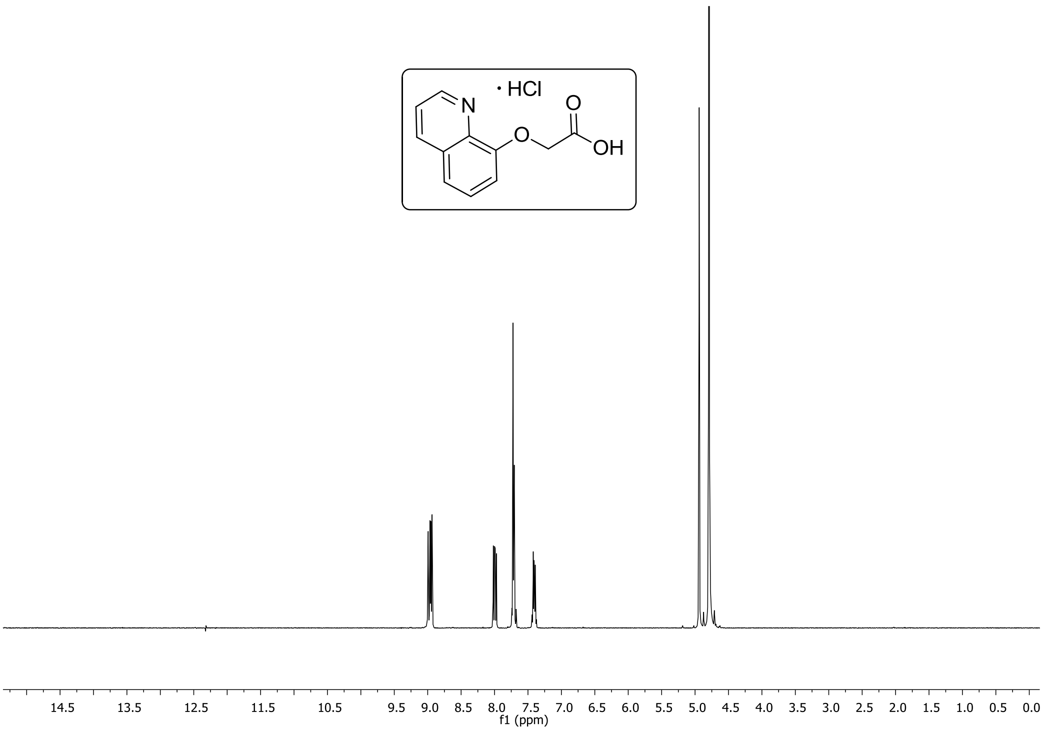

^{1}H NMR in D_2O (300 MHz): **Compound L11**.

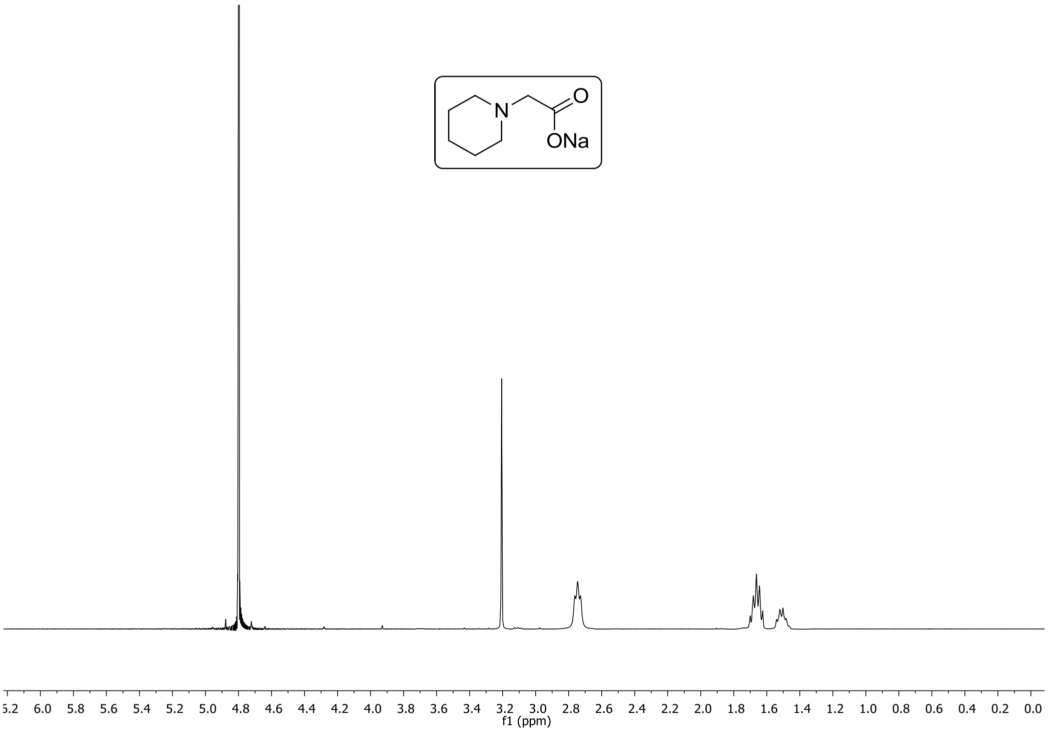

^{1}H NMR in D_2O (300 MHz): **Compound L15**.

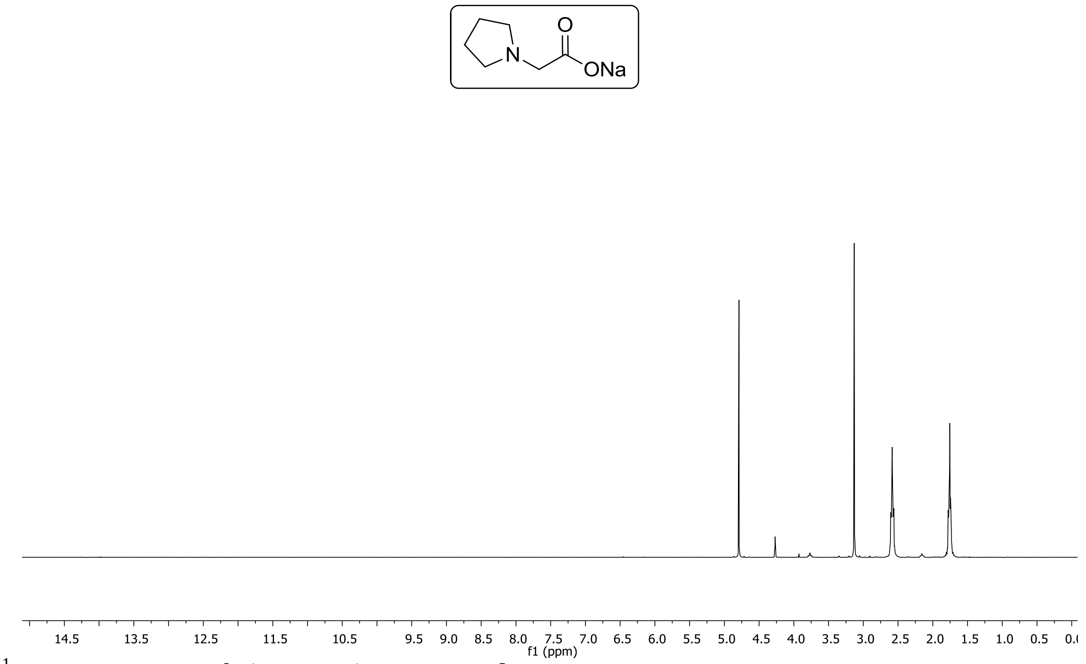

^{1}H NMR in DMSO-d_6 (300 MHz): **Compound L9**.

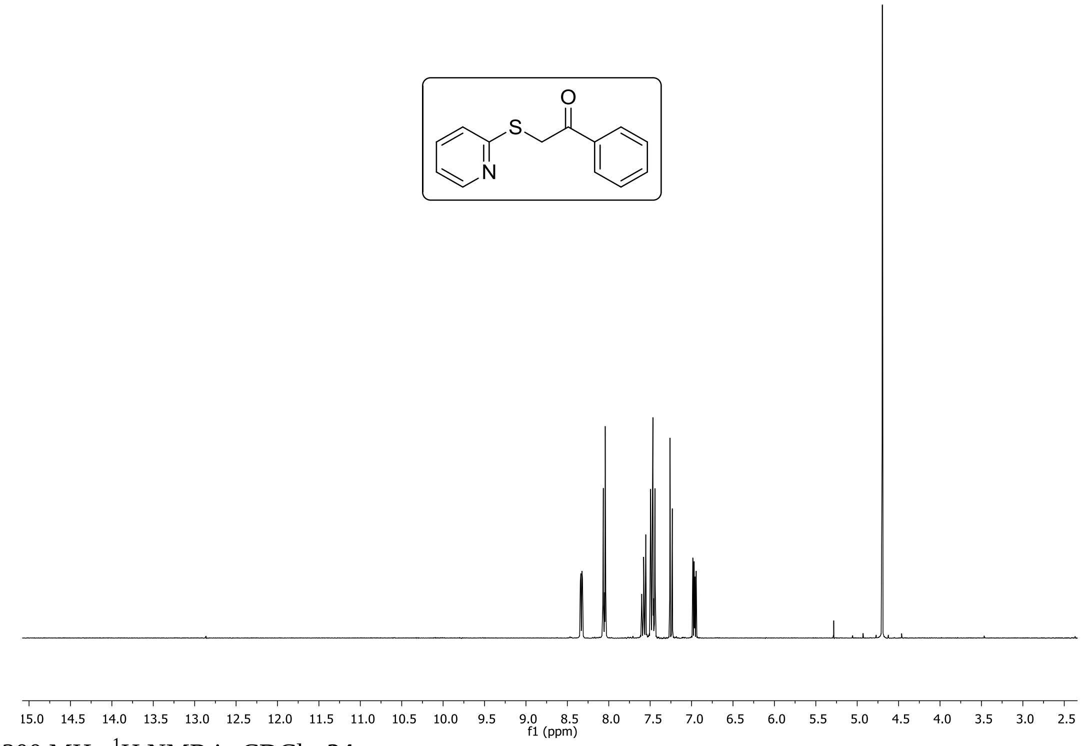

300 MHz-^{1}H NMR in $CDCl_3$: **24**.

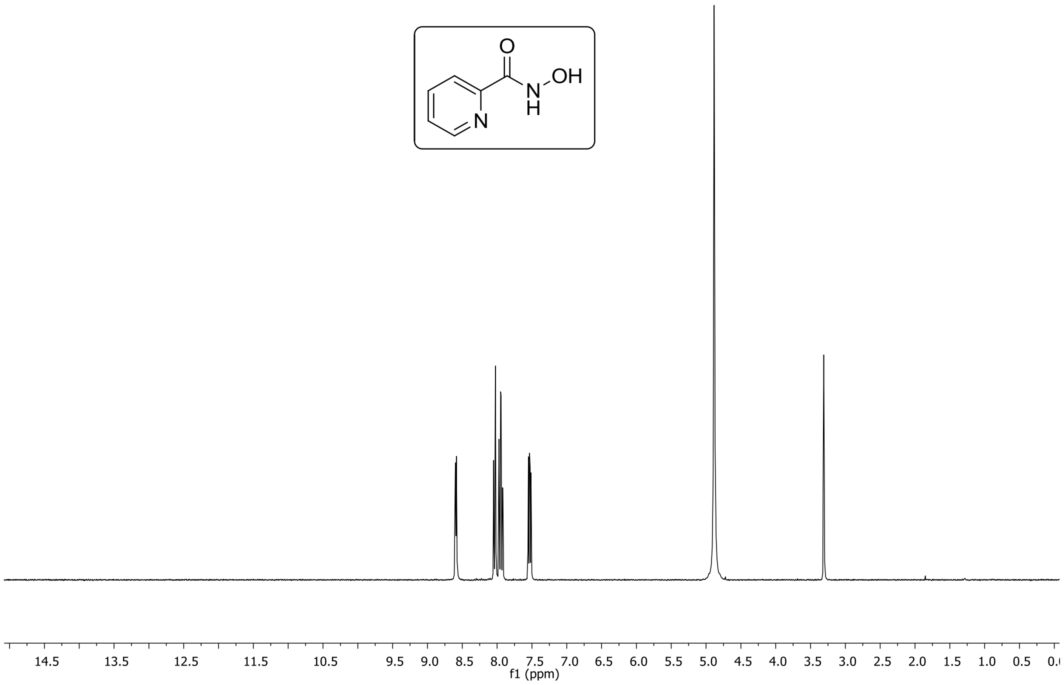

^{1}H NMR in MeOH-d_4 (300 MHz): **Compound L13**.

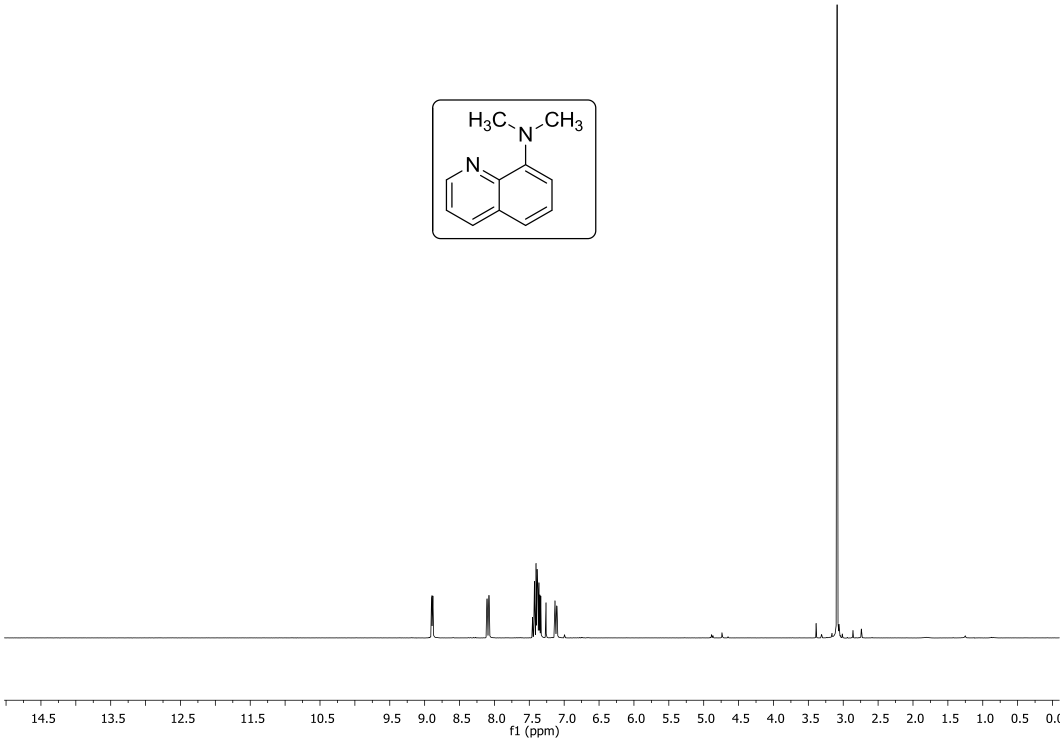

^{1}H NMR in $CDCl_3$ (300 MHz): **Compound L43**.

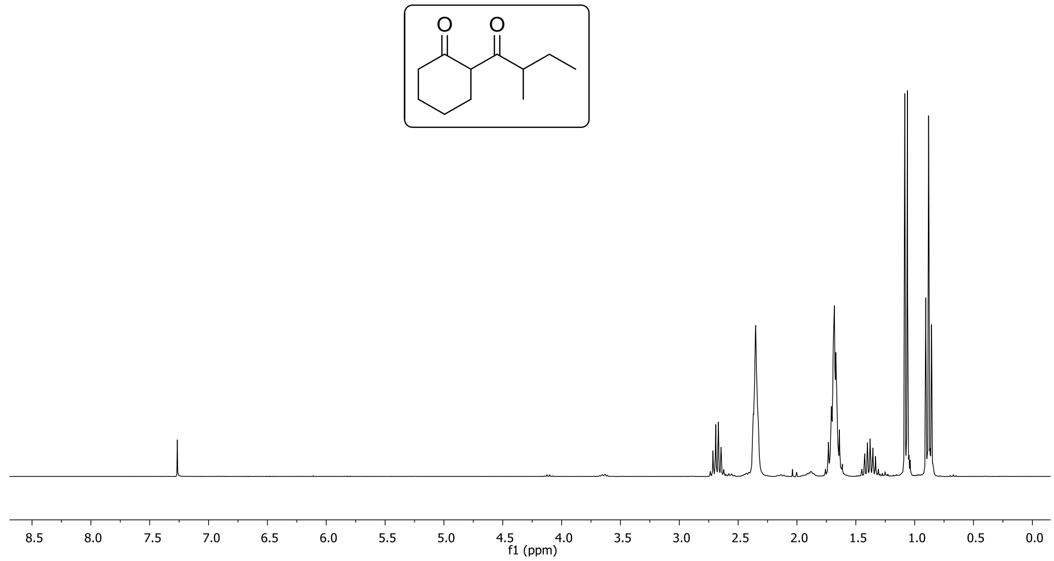

^{1}H NMR in $CDCl_3$ (300 MHz): **Compound L50**.

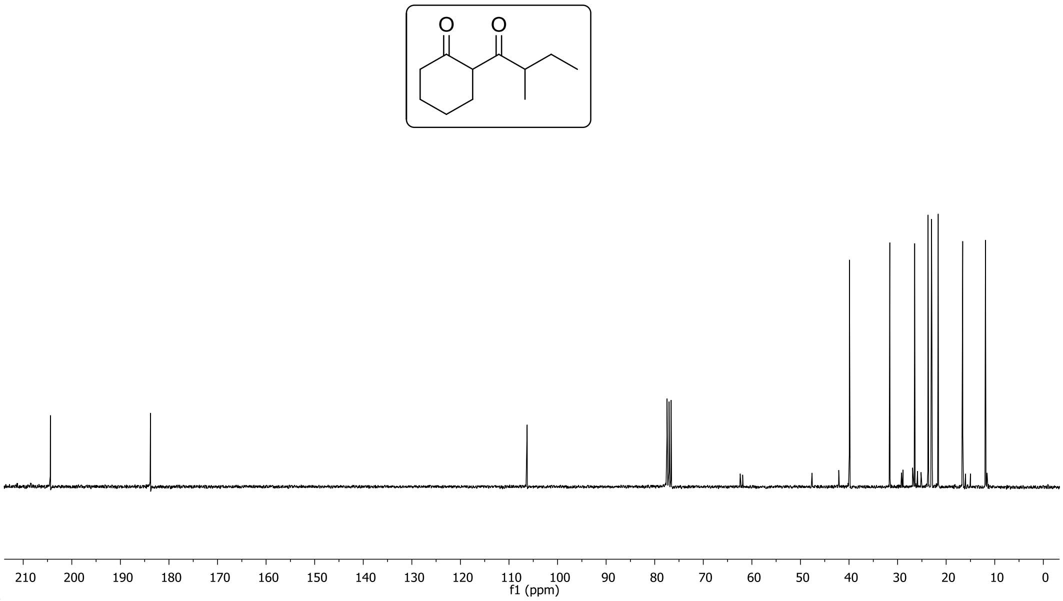

^{13}C NMR in $CDCl_3$ (75.5 MHz): **Compound L50**.

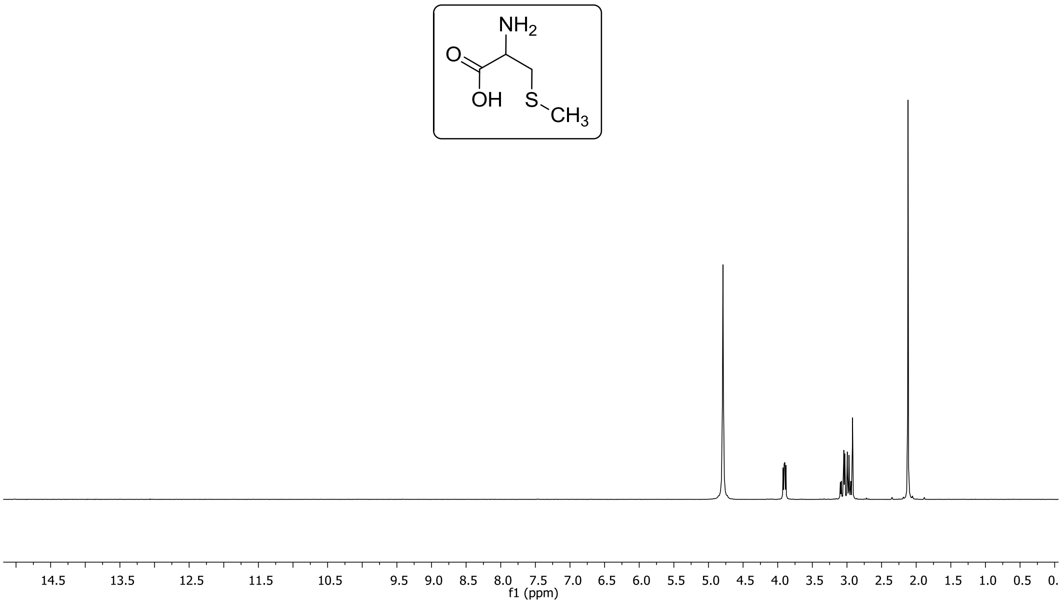

^{1}H NMR in D_2O (300 MHz): **Compound L8**.

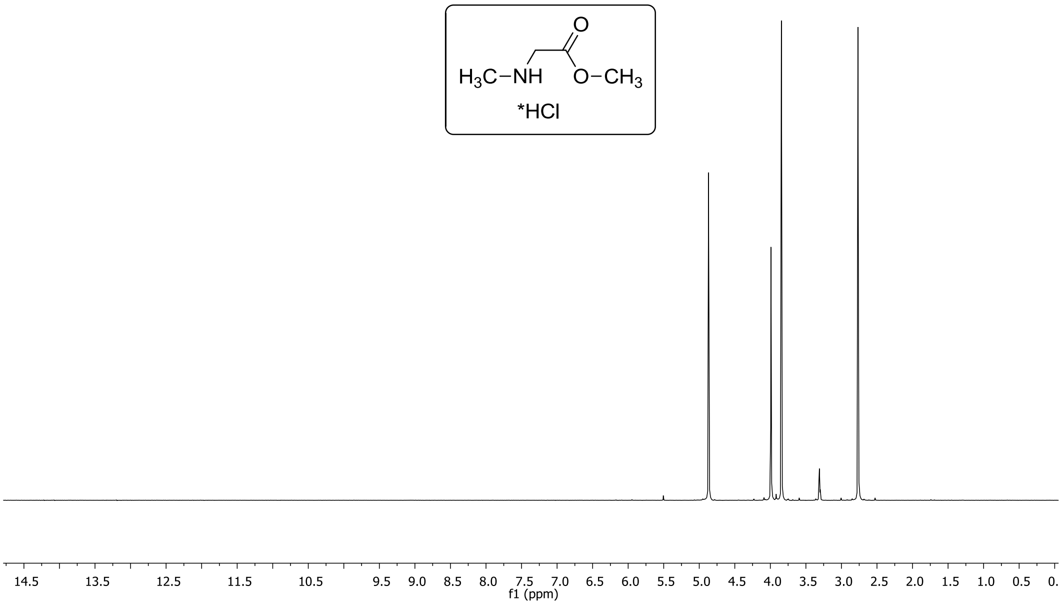

^{1}H NMR in MeOH-d_4 (300 MHz): **Compound 3.**

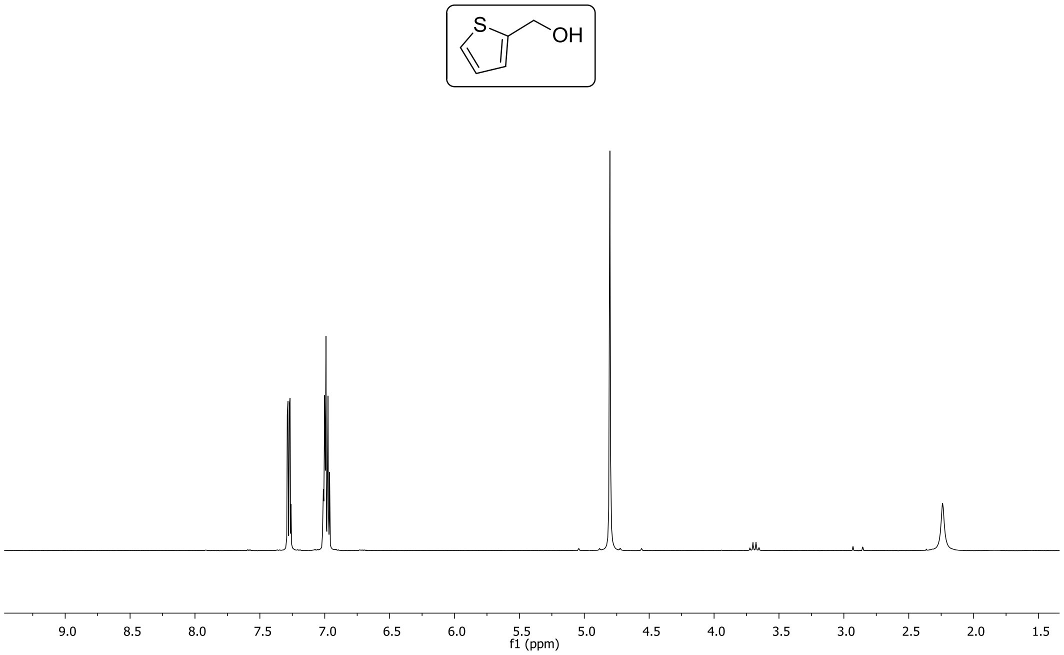

^{1}H NMR in $CDCl_3$ (300 MHz): **Compound 4**.

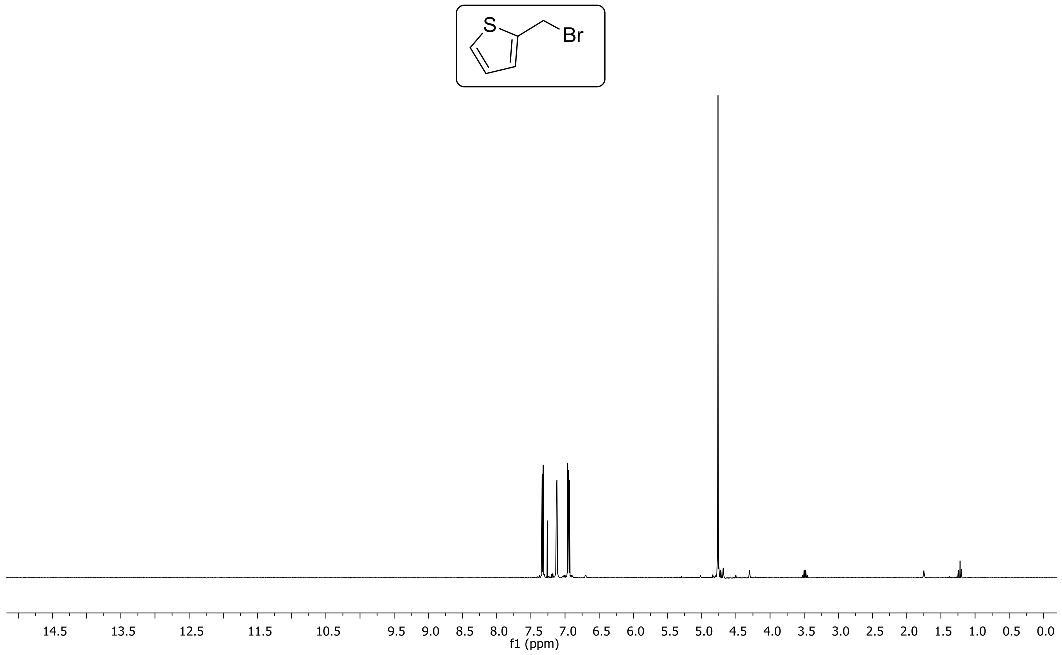

^{1}H NMR in $CDCl_3$ (300 MHz): **Compound 5**.

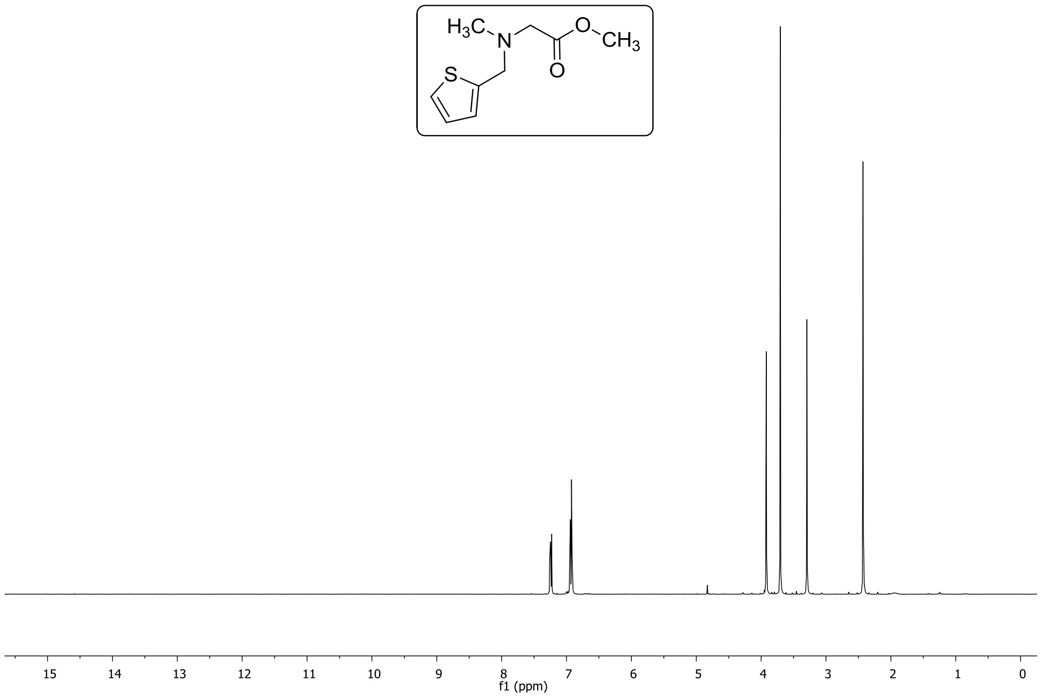

^{1}H NMR in $CDCl_3$ (300 MHz): **Compound 6**.

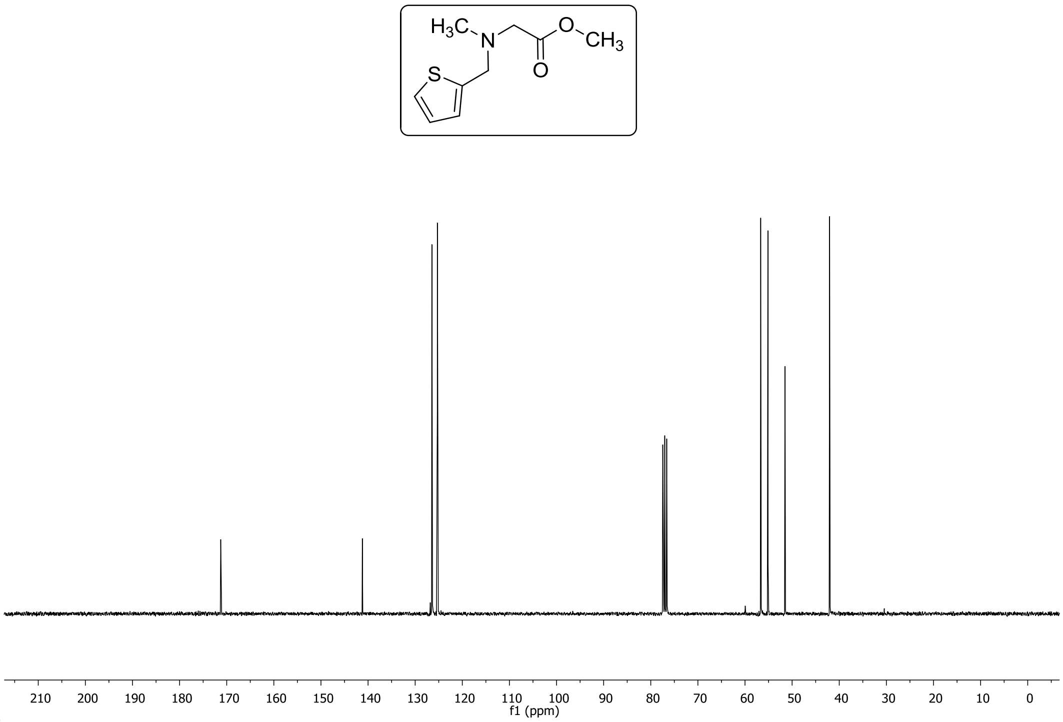

^{13}C NMR in $CDCl_3$ (75.5 MHz): **Compound 6**.

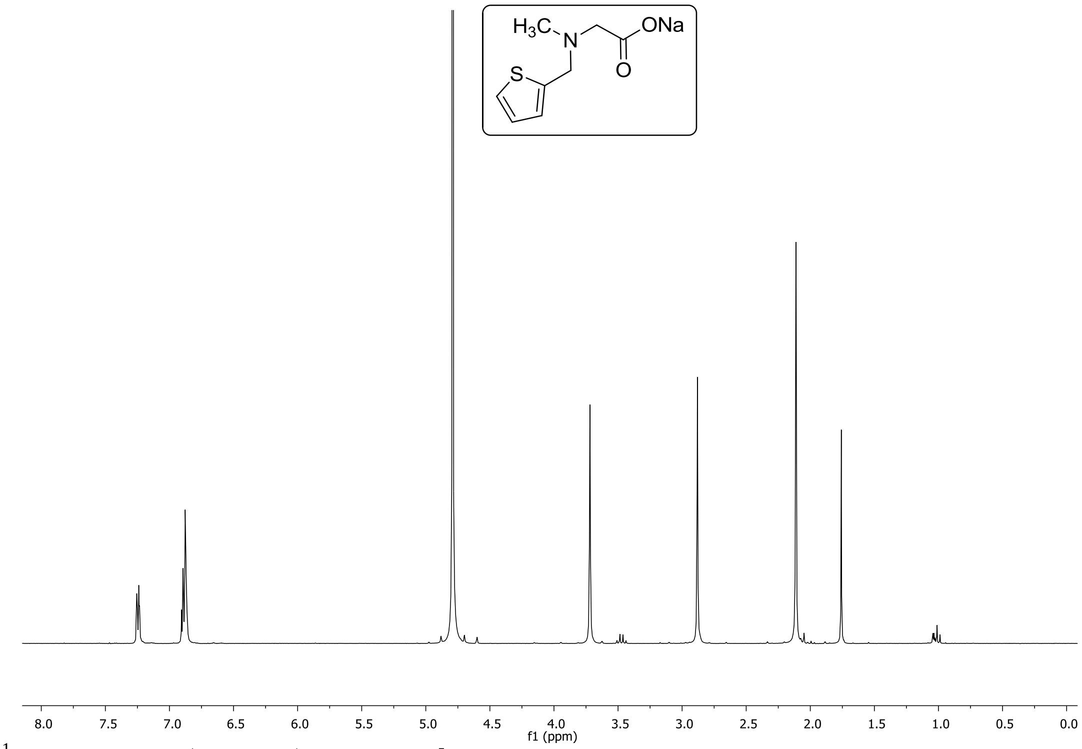

^{1}H NMR in D_2O (300 MHz): **Compound L18**.

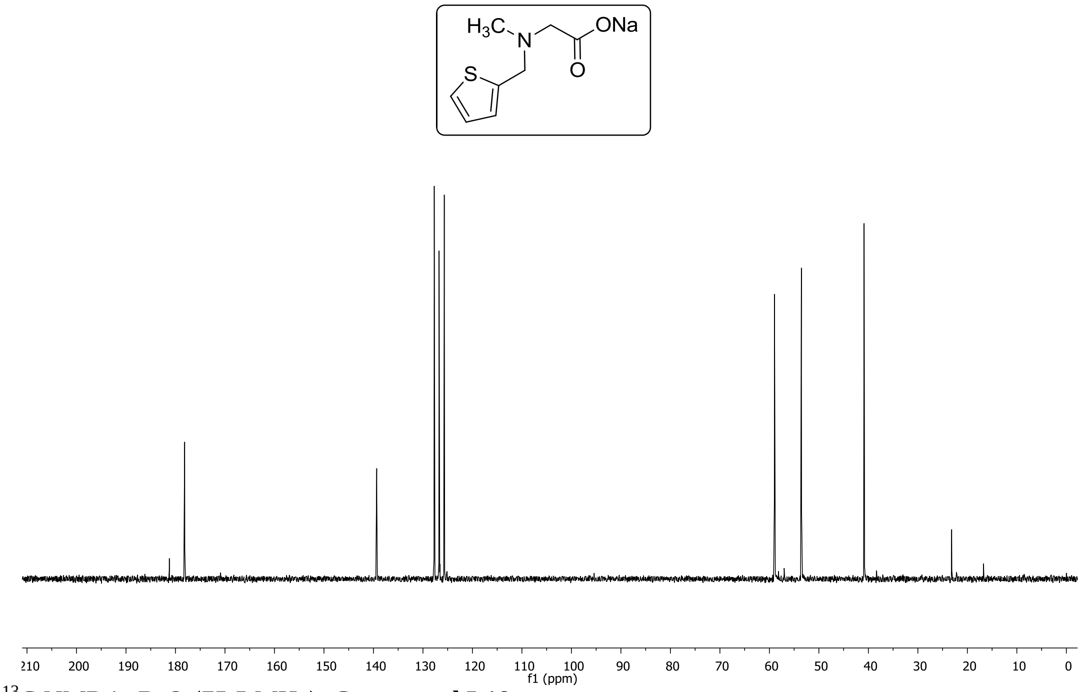

^{13}C NMR in D_2O (75.5 MHz): **Compound L18**.

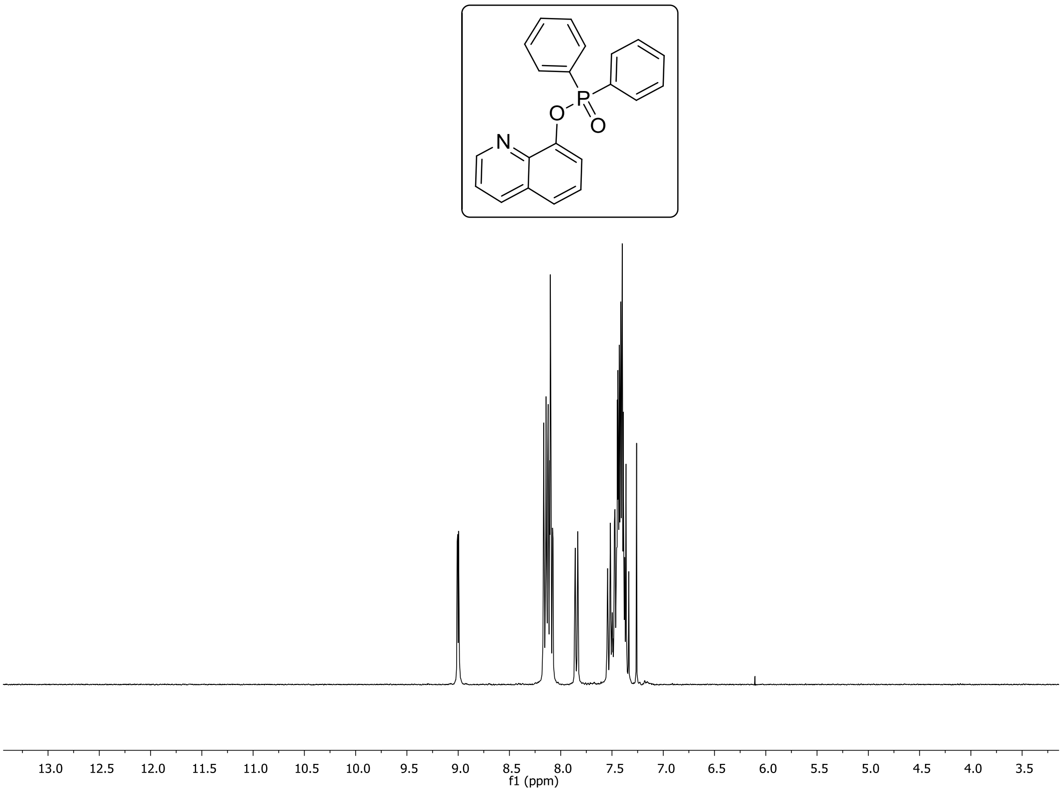

^{1}H NMR in $CDCl_3$ (300 MHz): **Compound L21**.

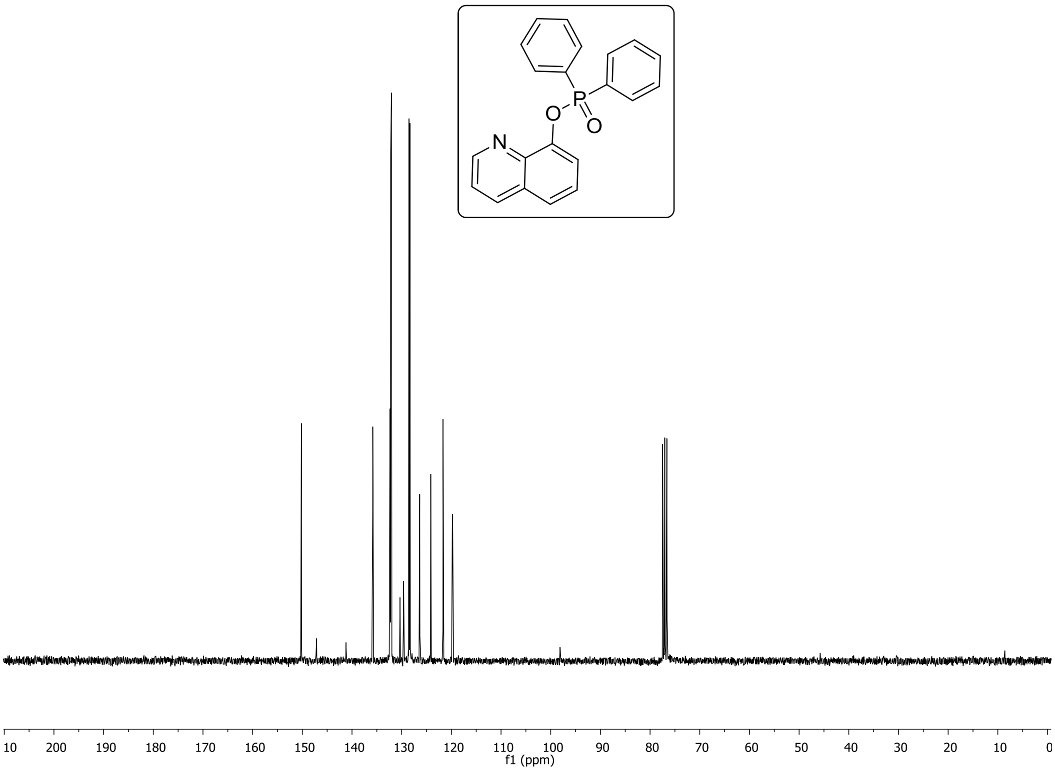

^{13}C NMR in $CDCl_3$ (75.5 MHz): **Compound L21**.

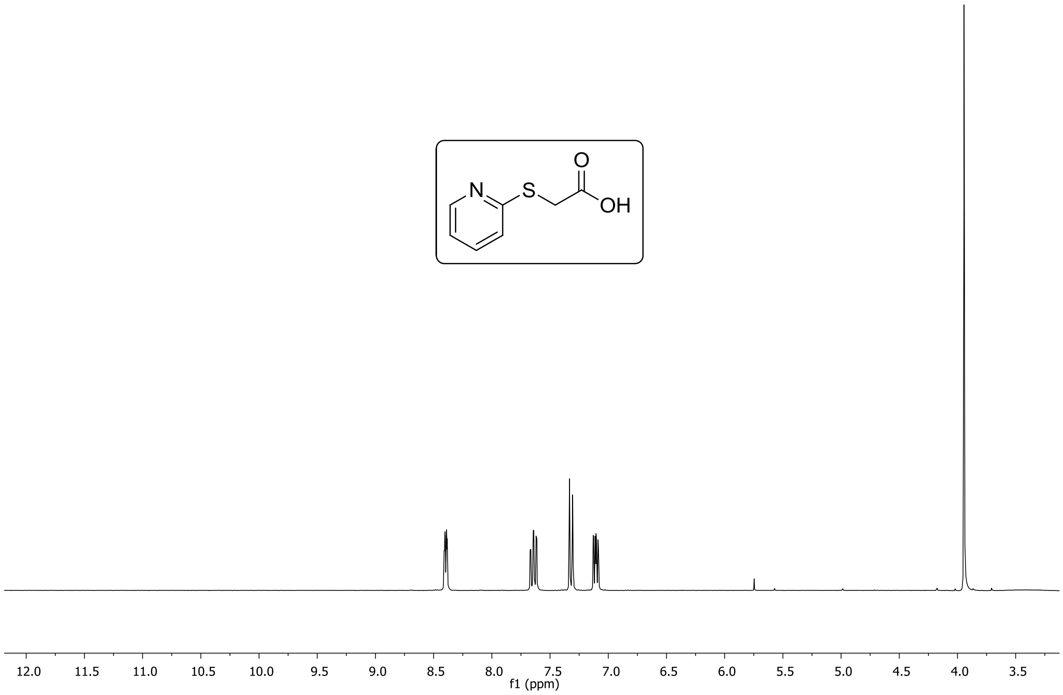

^1H NMR in DMSO-d_6 (300 MHz): **L10**.

References

[1] H. E. Gottlieb, V. Kotlyar, A. Nudelman, *J. Org. Chem.* **1997**, *62*, 7512-7515.

[2] H. J. Cristau, P. P. Cellier, S. Hamada, J. F. Spindler, M. Taillefer, *Org. Lett.* **2004**, *6*, 913-916.

[3] M. Taillefer, A. Ouali, J. F. Spindler, H. J. Cristau, *Adv. Synth. Catal.* **2006**, *348*, 499-505.

[4] Z. J. Song, E. Buck, D. Tschaen, P. G. Dormer, R. P. Volante, P. J. Reider, *Org. Lett.* **2002**, *4*, 1623-1626.

[5] D. C. Liotta, C. J. MacNevin, R. L. Moore, *J. Org. Chem.* **2008**, *73*, 1264-1269.

[6] E. Lukevics, E. Abele, R. Abele, J. Popelis, *Org. Prep. Proced. Int.* **2000**, *32*, 153-159.

[7] H. Feuer, D. M. Braunstein, *J. Org. Chem.* **1969**, *34*, 1817-1821.

[8] W. Kliegel, D. Nanninga, *Mon. Chem.* **1983**, *114*, 465-484.

[9] E. C. Wang, K. S. Huang, H. S. Chen, C. C. Wu, G. J. Lin, *J. Chin. Chem. Soc.* **2004**, *51*, 619-627.

[10] Z. Makles, O. V. Cebotarev, *Bull. Pol. Acad. Sci., Chem.* **1976**, *24*, 603.

[11] C. C. Hach, C. V. Banks, H. Diehl, *Org. Synth.* **1952**, *32*, 35-37.

[12] G. G. Mohamed, M. M. Omar, A. M. M. Hindy, *Spectrochim. Acta, Part A* **2005**, *62*, 1140-1150.

[13] R. R. Gallucci, *J. Chem. Eng. Data* **1982**, *27*, 217-219.

[14] J. Zhao, K. Cheng, Q. Z. Zheng, Y. Qian, L. Shi, H. L. Zhu, *Bioorg. Med. Chem.* **2009**, *17*, 7861-7871.

[15] M. K. Koley, P. T. Manoharan, A. P. Koley, *Inorg. Chim. Acta* **2010**, *363*, 3798-3802.

[16] I. Goldberg, G. K. Patra, *Eur. J. Inorg. Chem.* **2003**, 969-977.

[17] V. V. Kouznetsov, D. F. Amado, A. Bahsas, J. Amaro-Luis, *J. Heterocycl. Chem.* **2006**, *43*, 447-452.

[18] W. Langenbeck, G. Reinisch, K. Schönzart, *Chem. Ber.* **1959**, *92*, 2040-2042.

[19] H. T. Clarke, H. B. Gillespie, S. Z. Weisshaus, *J. Am. Chem. Soc.* **1933**, *55*, 4571-4587.

[20] S. Rahal, L. Badache, *Tetrahedron Lett.* **1991**, *32*, 3847-3848.

[21] L. Liu, X. Cui, Z. Li, C. Z. Tao, Y. Xu, J. Li, Q. X. Guo, *Org. Lett.* **2006**, *8*, 2467-2470.

[22] M. L. Rueppel, H. Rapoport, *J. Am. Chem. Soc.* **1972**, *94*, 3877-3883.

[23] A. T. Neffe, M. Bilang, B. Meyer, *Org. Biomol. Chem.* **2006**, *4*, 3259-3267.

[24] O. Nagel, *Mon. Chem.* **1897**, *18*, 31-43.

[25] M. Y. H. Lai, M. A. Brimble, D. J. Callis, P. W. R. Harris, M. S. Levi, F. Sieg, *Bioorg. Med. Chem.* **2005**, *13*, 533-548.

[26] I. Crosby, Avexa Limited, Patent WO 2007/124545A1 **2007**.

[27] A. E. Tschitschibabin, N. N. Woroshtzow, *Ber. Dtsch. Chem. Ges.* **1933**, *66*, 364-372.

[28] G. Tang, T. Ji, A. F. Hu, Y. F. Zhao, *Synlett* **2008**, 1907-1909.

[29] R. Baati, L. Louise-Leriche, E. Paunescu, G. Saint-Andre, A. Romieu, A. Wagner, P. Y. Renard, *Chem. Eur. J.* **2010**, *16*, 3510-3523.

[30] T. Tsukamoto, D. Ferraris, B. Duvall, Y. S. Ko, A. G. Thomas, C. Rojas, P. Majer, K. Hashimoto, *J. Med. Chem.* **2008**, *51*, 3357-3359.

[31] W. W. Paudler, J. E. Kuder, *J. Org. Chem.* **1967**, *32*, 2430-2433.

[32] C. Chen, K. M. Wilcoxen, C. Q. Huang, Y. F. Xie, J. R. McCarthy, T. R. Webb, Y. F. Zhu, J. Saunders, X. J. Liu, T. K. Chen, H. Bozigian, D. E. Grigoriadis, *J. Med. Chem.* **2004**, *47*, 4787-4798.

[33] S. Hünig, E. Benzing, E. Lücke, *Chem. Ber.* **1957**, *90*, 2833-2840.

[34] L. Liu, X. Cui, J. A. Li, Z. P. Zhang, Y. Fu, Q. X. Guo, *J. Org. Chem.* **2007**, *72*, 9342-9345.

[35] M. Selva, P. Tundo, A. Perosa, *J. Org. Chem.* **2003**, *68*, 7374-7378.

[36] M. Wlostowski, S. Czarnocka, P. Maciejewski, *Tetrahedron Lett.* **2010**, *51*, 5977-5979.

[37] H. Terayama, Mitsubishi Chemical Industries Limited, Patent US 4440788A1 **1984**.

[38] L. Goodman, L. O. Ross, B. R. Baker, *J. Org. Chem.* **1958**, *23*, 1251-1257.

[39] J. F. O'Connell, J. Parquette, W. E. Yelle, W. Wang, H. Rapoport, *Synthesis* **1988**, *1988*, 767,771.

[40] M. Rinken, W. D. Lehmann, W. A. Konig, *Liebigs Ann. Chem.* **1984**, 1672-1684.

[41] H. V. Huynh, Y. X. Chew, *Inorg. Chim. Acta* **2010**, *363*, 1979-1983.

[42] J. Langer, H. Görls, G. Gillies, D. Walther, *Zeitschr. Anorg. Allg. Chem.* **2005**, *631*, 2719-2726.
